# Phase plane analysis and novel soliton solutions for the space-time fractional Boussinesq equation using two robust techniques

**DOI:** 10.1371/journal.pone.0320190

**Published:** 2025-05-21

**Authors:** Tayyaba Younas, Jamshad Ahmad

**Affiliations:** Department of Mathematics, Faculty of Science, University of Gujrat, Gujrat, Pakistan; Tel Aviv University, ISRAEL

## Abstract

The Boussinesq equation is essential for studying the behavior of shallow water waves, surface waves in oceans and rivers, and the propagation of long waves in nonlinear systems. Its fractional form allows for a more accurate representation of wave dynamics by incorporating the effects of nonlocal interactions and memory. In this paper, we focus on obtaining exact traveling wave solutions for the space-time fractional Boussinesq equation using two well-established methods: the modified Sardar sub-equation method and the new extended direct algebraic method, both implemented with Atangana’s beta derivative. By applying these methods, we derive a variety of soliton solutions, including kink, anti-kink, periodic, dark, bright, and singular solitary waves. These solutions are presented in different mathematical forms, such as rational, hyperbolic, trigonometric, and exponential functions. This study not only provides new solutions but also enhances the understanding of wave propagation in fractional models, demonstrating the efficiency and applicability of the chosen methods. A comparative analysis of the methods and results is presented, along with an examination of the impact of fractional derivatives by adjusting their values. The study also includes 2D and 3D plots that illustrate the temporal behavior of the solutions. This study demonstrates that the methods employed are applicable to other nonlinear models in mathematical physics. A detailed analysis of the model’s behavior is conducted, focusing on bifurcation, chaos, and stability. Phase portrait analysis at critical points reveals shifts in the system’s dynamics, and introducing an external periodic force generates chaotic patterns. The solutions provided offer new insights into shallow water wave models, presenting effective tools for in-depth investigation of wave dynamics. All solutions are verified through MATHEMATICA and MATLAB simulations, ensuring their accuracy and reliability.

## 1 Introduction

Fractional calculus is a mathematical field that extends the concept of derivatives and integrals to non-integer, real, or even complex orders. This approach broadens traditional calculus, which typically deals with integer-order operations, allowing fractional calculus to model and analyze systems with memory, hereditary properties, or complex time-dependent behaviors. Initially introduced by mathematicians like Leibniz and later formalized by Abel in the 19th century, fractional calculus has gained significant interest in recent decades. It finds applications in diverse fields such as physics, biology, engineering, and economics, where systems often exhibit behaviors that change gradually or over time, making integer-based calculus insufficient. For instance, fractional calculus is used in viscoelasticity, signal processing, and diffusion processes, capturing dynamics that ordinary differential equations cannot. This flexibility and precision make fractional calculus a powerful tool for modeling complex, real-world phenomena in a more comprehensive way [1–3].

Nonlinear partial differential equations, developed as early as 1695, are essential tools for modeling various scientific phenomena in fields like biology, physics, circuit theory, and geochemistry. They often exhibit traveling wave solutions, which provide predictive insights into the future behavior of complex systems. Nonlinear equations are especially valuable in exploring wave dynamics across numerous applications, offering deeper understanding of system behaviors. Unlike their linear counterparts, nonlinear PDEs do not yield easily combined solutions, leading instead to unique phenomena such as shock waves, solitons, and chaotic behavior. Given their complexity, these equations typically require numerical methods or approximations for practical solutions [4–6]. Solitons are unique, stable wave forms that preserve their shape as they move without spreading out over time. Their stability comes from a delicate balance between nonlinearity and dispersion. This characteristic makes solitons important in areas such as fluid dynamics, fiber optics, and quantum mechanics, where they reveal essential insights into consistent wave behavior within complex systems [7, 8].

The fractional-order differential operator can be characterized in several ways, including the the Riemann-Liouville derivative [9], the Caputo fractional derivative [10], the conformable derivative [11], and Atangana’s beta derivative [12]. Among these, Atangana’s beta derivative has gained traction due to its successful applications across various scientific and engineering disciplines. This definition aligns closely with classical results of traditional derivatives, making it a valuable tool for researchers. In this study, we focus on the space-time fractional Boussinesq equation through the lens of the beta derivative, a concept introduced by Atangana and Baleanu in 2016. One of the advantages of Atangana’s beta derivative is its adherence to many properties characteristic of conventional derivatives. The modeling of real-world issues using fractional-order differential equations is essential for addressing a wide array of challenges in the physical sciences, highlighting the necessity for approximate, numerical, and exact solutions to these equations.

In response to the suggestion, we will provide a more detailed physical background of the equation studied. The Boussinesq equation is a fundamental model used to describe the behavior of shallow water waves, including wave propagation in oceans, rivers, and other similar systems. It captures important dynamics, such as nonlinearity and dispersion, and is used to model phenomena like long waves and solitary waves. The space-time fractional Boussinesq equation, as explored in this paper, incorporates fractional derivatives to better account for memory and hereditary effects in wave propagation, which is a more realistic representation of many natural systems. This extended form is particularly useful in modeling complex wave behaviors in various physical scenarios. By incorporating these derivatives, our study addresses the advanced dynamics of shallow water waves under fractional effects, providing a more comprehensive understanding of the model’s real-world applications. There has been increasing attention towards fractional differential equations due to their capability to accurately describe particle motion in complex processes and varied settings. One example is the fractional Boussinesq equation, which is applied to analyze water flow through porous media [13, 14]. El-Wakil and Abulwafa later advanced this work by developing the space-time fractional Boussinesq equation using fractional variational principles and the semi-inverse method [15]. This paper addresses the limited research on traveling wave solutions of the space-time fractional Boussinesq equation, highlighting its significance and the potential for diverse solutions. The study aims to utilize modified Sardar sub-equation method and the New extended direct algebraic method to derive these solutions. Ultimately, the goal is to deepen understanding and uncover various traveling wave solutions for this equation.

$$u_{tt}(x,t)+bu_{xx}(x,t)+s_{1}u_{xx}^{2}(x,t)+s_{2}u_{xxxx}(x,t)=0,$$ (1)

In this context, *u*(*x*,*t*) denotes the vertical displacement, while *b*, *s*_1_, and *s*_2_ are constant parameters. In recent years, several researchers have applied advanced methods to uncover diverse soliton solutions within fractional differential equations, particularly in the Boussinesq model. In 2024, M.A. Iqbal *et al*. utilized the (*G*′/(*G*′ + *G* + A))-expansion scheme with the beta derivative to produce distinct soliton types [16], while W. Razzaq and colleagues adopted both the F-expansion method and the modified (*G*′/(*G*′ + *G* + A))-expansion, revealing bright, dark, and periodic solitons [17]. M.M. Alzubaidi’s use of an improved tanh-function technique, coupled with Jumarie’s modified Riemann–Liouville derivative, resulted in hyperbolic, trigonometric, and rational soliton solutions [18]. Solutions to the perturbed Boussinesq equation emerged through the generalized projective Riccati equations method, yielding further insights [19]. In 2022, H. Chen’s application of the unified F-expansion technique uncovered unique periodic waveforms [20], and in 2021, H.Ç. Yaslan leveraged the conformable derivative for additional soliton types [21]. Similarly, K. Hosseini applied the modified Kudryashov method for soliton solutions [22], and M.A. Akbar’s team used the sine-Gordon expansion approach to derive bell-shaped, anti-bell-shaped, and kink solitons [23]. Together, these studies significantly advance the understanding of nonlinear wave propagation.

Analytical soliton solutions in fractional models are important because they help improve models, support various applications, and expand scientific knowledge in different fields. The shape and behavior of waves can change depending on certain values, especially influenced by the fractional parameter. This study looks at unique soliton solutions to the space–time fractional Boussinesq equations using the modified Sardar sub-equation method and new extended direct algebraic method, focusing on how the fractional derivative affects wave behavior as it increases.

This paper uses the modified Sardar subequation method to solve a complex model by breaking down difficult nonlinear equations into simpler parts, leading to soliton solutions. The method produces various solutions, including trigonometric, hyperbolic, and exponential types, though it can be time-consuming. This research offers new insights into nonlinear equations and contributes to a deeper theoretical understanding in this area. The new extended algebraic method brings versatility and valuable insights to solving a range of mathematical problems, and it can even enhance computational efficiency in some cases. While particularly effective for certain types of equations, the complexity of its derivations can be challenging. This method is especially helpful for finding alternative solutions to difficult problems where traditional approaches may fall short, though its success depends on specific parameters and assumptions. Despite its potential to generate various solutions, the method may also require a significant time investment.

Several mathematical models have been effectively solved using the modified Sardar subequation method and the new extended algebraic method, including nonlinear Schrödinger equation [24], Kudryashov’s equation [25], 4th-order (2+ 1)-dimensional Schrödinger equation [26], time-fractional perturbed Fokas–Lenells equation [27], fractional coupled Boussinesq model [28], Tzitzica type evolution equations [42], resonant nonlinear Schrödinger equation [29], Zoomeron equation [30], Zakhrov equation [31], Kuralay equation [32], Kairat-X equation [33] etc.

The manuscript is organized as follows: Sect 2 presents an introduction to Atangana’s beta derivative. Sect 3 contains the detail of Boussinesq equation. In Sect 4, we provide an overview of the modified Sardar sub-equation method and the new extended direct algebraic method. Sect 5 details the process of obtaining solutions for the proposed model. Sect 6 discusses the graphs of the results, while Sect 7 examines the system’s dynamic behavior through phase plane analysis. Finally, Sect 8 concludes with closing remarks.

## 2 Atangana’s beta derivative

To introduce the concept of the beta derivative, we first define the conformable fractional derivative. This derivative, initially proposed by Khalil *et al*. [34] and further refined by Abdeljawad [35], provides a foundation for fractional calculus. The conformable fractional derivative of a function F:[0,∞) with respect to the order *α* is defined as follows:


$${\;}^{0}D_{x}^{\alpha }[F(x)]=\underset{\delta \to 0}{\lim}\frac{F(x+\delta x^{1-\alpha })-F(x)}{\delta },\;t>0,0<\alpha <1.$$

If *F* is *α*-differentiable in the range (0,b), where *b*>0, and $\lim_{\delta \to 0^{+}}F^{\left(\alpha \right)}(x)$ exists, then we define


$$F^{\left(\alpha \right)}(0)=\lim_{\delta \to 0^{+}}F^{\left(\alpha \right)}(x).$$

The conformable fractional derivative has distinct characteristics compared to the classical derivative, leading to the need for a refined approach. To align more closely with traditional calculus, the definition is extended to what is known as the beta derivative. This derivative, as outlined in [36], aims to bridge the gap by offering a more consistent framework that preserves key properties of classical derivatives while accommodating the flexibility of fractional calculus. The beta derivative’s formulation is provided as follows:

$${\;}_{0}^{A}D_{x}^{\alpha }[F(x)]=\lim_{\delta \to 0}\frac{F\left(x+\delta {\left(x+\frac{1}{\Gamma (\alpha )}\right)}^{\frac{1}{\alpha }}\right)-F(x)}{\delta }.$$ (2)

This study examines a model based on Atangana’s beta derivative to highlight the essential properties of fundamental derivative rules. By utilizing this derivative, the research enhances the understanding of complex behaviors in various mathematical and scientific applications.

**Theorem.** Assume that *u* and *v* are two *α*-differentiable functions of *t* where 0<α≤1 and α>0, the following conclusions are drawn from the definition of beta derivative:

1. ${\;}_{A}^{0}D_{t}^{\alpha }(lu(t)+mw(t))=l_{A}^{0}D_{t}^{\alpha }u(t)+m_{A}^{0}D_{t}^{\alpha }w(t)$,
2. ${\;}_{A}^{0}D_{t}^{\alpha }(u(t)*w(t))=w(t{)}_{A}^{0}D_{t}^{\alpha }u(t)+u(t{)}_{A}^{0}D_{t}^{\alpha }w(t)$,
3. ${\;}_{A}^{0}D_{t}^{\alpha }\left(\frac{u(t)}{w(t)}\right)=\frac{w(t)D_{t}^{\alpha }u(t)-u(t)D_{t}^{\alpha }w(t)}{v(t){\;}^{2}}$,
4. ${\;}_{A}^{0}D_{t}^{\alpha }(C)=0$, where *C* is constant,
5. ${\;}_{A}^{0}D_{t}^{\alpha }(u(t))=(t+\frac{1}{\Gamma (\alpha )}){\;}^{1-\alpha }\frac{d}{dt}\{u(t)\}$.

## 3 Governing equation

In this section, we explore the space-time fractional Boussinesq equation, incorporating beta fractional derivatives.

Eq 1 utilizing the beta derivative is expressed as follows:

$$d{\;}_{A}^{0}D_{t}^{2\alpha }u(x,t)+{\;}_{A}^{0}D_{x}^{2\alpha }u(x,t)+s_{1}\;{\;}_{A}^{0}D_{x}^{2\alpha }u^{2}(x,t)+s_{2}\;{\;}_{A}^{0}D_{x}^{4\alpha }u(x,t)=0.$$ (3)

Here, ${\;}_{A}^{0}D_{x}^{\alpha }u$ and ${\;}_{A}^{0}D_{t}^{\alpha }u$ refer to the beta derivatives of *u* with respect to *x* and *t* respectively.

For beta derivative, we use the following wave transformation

$$u(x,t)=v(\xi ),\;\;\;\xi =\frac{k{\left(\frac{1}{\Gamma (\alpha )}+x\right)}^{\alpha }}{\alpha }+\frac{\omega {\left(\frac{1}{\Gamma (\alpha )}+t\right)}^{\alpha }}{\alpha }.$$ (4)

Here, *k* denotes the coefficient of the spatial variable, while ω represents the speed of the traveling wave.

By applying the wave transformation, we obtain the following expressions.

$$(\omega^{2}+bk^{2})\nu^{\prime \prime }+2k^{2}s_{2}{\left(\nu^{2}\right)}^{\prime \prime }+k^{4}s_{1}\nu^{\prime \prime \prime \prime \prime }=0,$$ (5)

which can be integrated twice and simplify to yield

$$(bk^{2}+\omega^{2})v+k^{2}s_{2}v^{2}+k^{4}s_{1}v^{\prime \prime }=0.$$ (6)

Here, the integration constants are set to zero.

## 4 Summary of methods

We begin by presenting the foundational steps of both methods. The analysis initiates with the following nonlinear partial differential equation.

$$N(u,u_{x},u_{t},u_{xx},u_{tt},u_{xt},\ldots )=0.$$ (7)

In this setup, u(x,t) represents an unknown function that depends on both *x* and *t*, with partial derivatives indicating its rate of change in each direction. By applying a wave transformation, we reduce this partial differential equation to an ordinary differential equation, simplifying the process of finding solutions.

$$U(u,u^{'},u^{''},u^{'''},...)=0.$$ (8)

### Modified Sardar sub-equation method

In this section, we present an enhancement of the Sardar sub-equation method, specifically designed to derive a diverse set of new solutions for the space-time fractional Boussinesq equation.

The resulting series solutions for Eq 6 can be expressed as follows:

$$v(\xi )=a_{0}+\sum_{j=1}^{N}a_{j}F^{j}(\xi ),$$ (9)

Here, $a_{0},a_{1},a_{2},\ldots$ represent constants. To determine the value of *N*, we use the balancing principle. Furthermore, the function F(ξ) is required to satisfy the following equation:

$$(F^{\prime }(\xi ){)}^{2}=\beta F(\xi {)}^{2}+\delta F(\xi {)}^{4}+\Omega .$$ (10)

where Ω, β, and δ are constants. Next, we present the solutions that include the parameter σ as follows:

**Family 1:** If δ≠0 , β>0 and Ω=0 ,then


$$F_{1}^{\pm }(\xi )=\pm \sqrt{-\frac{\beta }{\delta }}\text{sech}\left(\sqrt{\beta }(\xi +\sigma )\right),$$

$$F_{2}^{\pm }(\xi )=\pm \sqrt{\frac{\beta }{\delta }}\text{csch}\left(\sqrt{\beta }(\xi +\sigma )\right).$$

**Family 2:** If $\delta =\pm E_{1}E_{2}$ , β>0 and Ω=0 ,then


$$F_{3}^{\pm }(\xi )=\pm \frac{e_{1}\sqrt{\beta }}{\left(4e_{1}^{2}-\delta \right)\sinh\left(\sqrt{\beta }(\xi +\sigma )\right)+\left(4e_{1}^{2}-\delta \right)\cosh\left(\sqrt{\beta }(\xi +\sigma )\right)}.$$

Where *E*_1_ and *E*_2_ are real constant.

**Family 3:** If δ>0 , β<0 and $\Omega =\frac{\beta^{2}}{4\delta }$ ,then following solutions are obtained


$$F_{4}^{\pm }(\xi )=\pm \sqrt{-\frac{\beta }{2\delta }}\tanh\left(\sqrt{-\frac{\beta }{2}}(\xi +\sigma )\right),$$

$$F_{5}^{\pm }(\xi )=\pm \sqrt{-\frac{\beta }{2\delta }}\coth\left(\sqrt{-\frac{\beta }{2}}(\xi +\sigma )\right),$$

$$F_{6}^{\pm }(\xi )=\sqrt{-\frac{\beta }{2\delta }}\left(\tanh\left(\sqrt{-2\beta }(\xi +\sigma )\right)+i\text{sech}\left(\sqrt{-2\beta }(\xi +\sigma )\right)\right),$$

$$F_{7}^{\pm }(\xi )=\sqrt{-\frac{\beta }{8\delta }}\left(\tanh\left(\sqrt{-\frac{\beta }{8}}(\xi +\sigma )\right)+i\coth\left(\sqrt{-\frac{\beta }{8}}(\xi +\sigma )\right)\right),$$

$$F_{8}^{\pm }(\xi )=\pm \frac{\sqrt{-\frac{\beta }{2\delta }}\left(\sqrt{W_{1}^{2}+W_{2}^{2}}-e_{1}\cosh\left(\sqrt{-2\beta }(\xi +\sigma )\right)\right)}{F_{2}+W_{1}\sinh\left(\sqrt{-2\beta }(\xi +\sigma )\right)},$$

Where *F*_1_ and *F*_2_ are real constants.


$$F_{9}^{\pm }(\xi )=\pm \frac{\sqrt{-\frac{\beta }{2\delta }}\cosh\left(\sqrt{-2\beta }(\xi +\sigma )\right)}{\sinh\left(\sqrt{-2\beta }(\xi +\sigma )\right)+i}.$$

**Family 4:** If δ≠0 , β<0 and Ω=0 ,then following solutions are obtained


$$F_{10}^{\pm }(\xi )=\pm \sqrt{-\frac{\beta }{\delta }}\sec\left(\sqrt{-\beta }(\xi +\sigma )\right),$$

$$F_{11}^{\pm }(\xi )=\pm \sqrt{-\frac{\beta }{\delta }}\csc\left(\sqrt{-\beta }(\xi +\sigma )\right).$$

**Family 5:** If δ>0 , β>0 and $\Omega =\frac{\beta^{2}}{4\delta }$ and $F_{1}^{2}-F_{2}^{2}>0$ ,we get


$$F_{12}^{\pm }(\xi )=\pm \sqrt{\frac{\beta }{2\delta }}\tan\left(\sqrt{\frac{\beta }{2}}(\xi +\sigma )\right),$$

$$F_{13}^{\pm }(\xi )=\pm \sqrt{\frac{\beta }{2\delta }}\cot\left(\sqrt{\frac{\beta }{2}}(\xi +\sigma )\right),$$

$$F_{14}^{\pm }(\xi )=\pm \sqrt{\frac{\beta }{2\delta }}\left(\tan\left(\sqrt{2\beta }(\xi +\sigma )\right)+\sec\left(\sqrt{2\beta }(\xi +\sigma )\right)\right),$$

$$F_{15}^{\pm }(\xi )=\pm \sqrt{\frac{\beta }{8\delta }}\left(\tan\left(\sqrt{\frac{\beta }{8}}(\xi +\sigma )\right)-\cot\left(\sqrt{\frac{\beta }{8}}(\xi +\sigma )\right)\right),$$

$$F_{16}^{\pm }(\xi )=\pm \frac{\sqrt{\frac{\beta }{2\delta }}\left(\sqrt{W_{1}^{2}-W_{2}^{2}}-W_{1}\cos\left(\sqrt{2\beta }(\xi +\sigma )\right)\right)}{F_{2}+W_{1}\sin\left(\sqrt{2\beta }(\xi +\sigma )\right)},$$

$$F_{17}^{\pm }(\xi )=\pm \frac{\sqrt{\frac{\beta }{2\delta }}\cos\left(\sqrt{2\beta }(\xi +\sigma )\right)}{\sin\left(\sqrt{2\beta }(\xi +\sigma )\right)+1}.$$

**Family 6:** If β>0 and Ω=0,we get


$$F_{18}^{\pm }(\xi )=\pm \frac{4\beta e^{\left(\sqrt{\beta }(\xi +\sigma )\right)}}{e^{\left(2\sqrt{\beta }(\xi +\sigma )\right)-4\beta \delta }},$$

$$F_{19}^{\pm }(\xi )=\pm \frac{4\beta e^{\left(\sqrt{\beta }(\xi +\sigma )\right)}}{1-4\beta \delta e^{\left(2\sqrt{\beta }(\xi +\sigma )\right)}}.$$

**Family 7:** If δ>0 and β=Ω=0,we get


$$F_{20}^{\pm }(\xi )=\pm \frac{1}{\sqrt{\delta }(\xi +\sigma )}.$$

**Family 8:** If δ<0 and β=Ω=0,we get


$$F_{21}^{\pm }(\xi )=\pm \frac{i}{\sqrt{-\delta }(\xi +\sigma )}.$$

### 4.1 New extended direct algebraic method

By employing the previously mentioned transformation, we can solve Eq 1 effectively. This approach simplifies the equation, allowing us to derive its solutions and gain a deeper understanding of its implications and behavior.

$$u(\xi )=\sum_{j=0}^{N}a_{j}F^{j}(\xi ),$$ (11)

$$F^{\prime }(\xi )=\ln(A)\left(\gamma +\beta F(\xi )+\mu F(\xi {)}^{2}\right).$$ (12)

In this context, γ, β, and μ represent constants. Listed below are specific solutions derived for Eq 6:

**Family 1**: If $\beta^{2}-\gamma \mu <0$ and μ≠0, then


$$F_{1}(\xi )=-\frac{\beta }{2\mu }+\frac{\sqrt{-\left(\beta^{2}-4\gamma \mu \right)}}{2\mu }\tan_{A}\left(\frac{\sqrt{-\left(\beta^{2}-4\gamma \mu \right)}}{2}\xi \right),$$

$$F_{2}(\xi )=-\frac{\beta }{2\mu }+\frac{\sqrt{-\left(\beta^{2}-4\gamma \mu \right)}}{2\mu }\cot_{A}\left(\frac{\sqrt{-\left(\beta^{2}-4\gamma \mu \right)}}{2}\xi \right),$$

$$F_{3}(\xi )=-\frac{\beta }{2\mu }+\frac{\sqrt{-\left(\beta^{2}-4\gamma \mu \right)}}{2\mu }\left({\text{tan}}_{A}\left(\sqrt{-\left(\beta^{2}-4\gamma \mu \right)}\xi \right)\pm \sqrt{\text{pq}}\;{\text{sec}}_{A}\left(\sqrt{-\left(\beta^{2}-4\gamma \mu \right)}\xi \right)\right),$$

$$F_{4}(\xi )=-\frac{\beta }{2\mu }+\frac{\sqrt{-\left(\beta^{2}-4\gamma \mu \right)}}{2\mu }\left({\text{-cot}}_{A}\left(\sqrt{-\left(\beta^{2}-4\gamma \mu \right)}\xi \right)\pm \sqrt{\text{pq}}\;{\text{csc}}_{A}\left(\sqrt{-\left(\beta^{2}-4\gamma \mu \right)}\xi \right)\right),$$

$$F_{5}(\xi )=-\frac{\beta }{2\mu }+\frac{\sqrt{-\left(\beta^{2}-4\gamma \mu \right)}}{2\mu }\left({\text{tan}}_{A}\left(\frac{\sqrt{-\left(\beta^{2}-4\gamma \mu \right)}}{4}\xi \right)-{\text{cot}}_{A}\left(\frac{\sqrt{-\left(\beta^{2}-4\gamma \mu \right)}}{4}\xi \right)\right).$$

**Family 2**: If $\beta^{2}-\gamma \mu >0$ and μ≠0, then


$$F_{6}(\xi )=-\frac{\beta }{2\mu }+\frac{\sqrt{\left(\beta^{2}-4\gamma \mu \right)}}{2\mu }\tanh_{A}\left(\frac{\sqrt{\left(\beta^{2}-4\gamma \mu \right)}}{2}\xi \right),$$

$$F_{7}(\xi )=-\frac{\beta }{2\mu }+\frac{\sqrt{\left(\beta^{2}-4\gamma \mu \right)}}{2\mu }\coth_{A}\left(\frac{\sqrt{\left(\beta^{2}-4\gamma \mu \right)}}{2}\xi \right),$$

$$F_{8}(\xi )=-\frac{\beta }{2\mu }+\frac{\sqrt{\left(\beta^{2}-4\gamma \mu \right)}}{2\mu }\left(-{\text{tanh}}_{A}\left(\sqrt{\left(\beta^{2}-4\gamma \mu \right)}\xi \right)\pm i\sqrt{\text{pq}}\;{\text{sech}}_{A}\left(\sqrt{\left(\beta^{2}-4\gamma \mu \right)}\xi \right)\right),$$

$$F_{9}(\xi )=-\frac{\beta }{2\mu }+\frac{\sqrt{\left(\beta^{2}-4\gamma \mu \right)}}{2\mu }\left({\text{-coth}}_{A}\left(\sqrt{\left(\beta^{2}-4\gamma \mu \right)}\xi \right)\pm \sqrt{\text{pq}}\;{\text{csch}}_{A}\left(\sqrt{\left(\beta^{2}-4\gamma \mu \right)}\xi \right)\right),$$

$$F_{10}(\xi )=-\frac{\beta }{2\mu }+\frac{\sqrt{\left(\beta^{2}-4\gamma \mu \right)}}{2\mu }\left({\text{tanh}}_{A}\left(\frac{\sqrt{\left(\beta^{2}-4\gamma \mu \right)}}{4}\xi \right)\pm \sqrt{\text{pq}}\;{\text{coth}}_{A}\left(\frac{\sqrt{\left(\beta^{2}-4\gamma \mu \right)}}{4}\xi \right)\right).$$

**Family 3**: If γμ>0 and β=0 , then


$$F_{11}(\xi )=\sqrt{\frac{\gamma }{\mu }}{\text{tan}}_{A}\left(\xi \sqrt{\gamma \mu }\right),$$

$$F_{12}(\xi )=-\sqrt{\frac{\gamma }{\mu }}{\text{cot}}_{A}\left(\xi \sqrt{\gamma \mu }\right),$$

$$F_{13}(\xi )=\sqrt{\frac{\gamma }{\mu }}\left({\text{tan}}_{A}\left(2\xi \sqrt{\gamma \mu }\right)\pm \sqrt{\text{pq}}\;{\text{sec}}_{A}\left(2\xi \sqrt{\gamma \mu }\right)\right),$$

$$F_{14}(\xi )=\sqrt{\frac{\gamma }{\mu }}\left(-{\text{cot}}_{A}\left(2\xi \sqrt{\gamma \mu }\right)\pm \sqrt{\text{pq}}\;{\text{csc}}_{A}\left(2\xi \sqrt{\gamma \mu }\right)\right),$$

$$F_{15}(\xi )=\frac{1}{2}\sqrt{\frac{\gamma }{\mu }}\left({\text{tan}}_{A}\left(2\xi \frac{\sqrt{\gamma \mu }}{2}\right)-{\text{cot}}_{A}\left(2\xi \frac{\sqrt{\gamma \mu }}{2}\right)\right).$$

**Family 4**: If γμ<0 and β=0 , then


$$F_{16}(\xi )=-\sqrt{\frac{-\gamma }{\mu }}{\text{tanh}}_{A}\left(\xi \sqrt{-\gamma \mu }\right),$$

$$F_{17}(\xi )=-\sqrt{\frac{-\gamma }{\mu }}{\text{coth}}_{A}\left(\xi \sqrt{-\gamma \mu }\right),$$

$$F_{18}(\xi )=\sqrt{\frac{-\gamma }{\mu }}\left(-{\text{tanh}}_{A}\left(2\xi \sqrt{-\gamma \mu }\right)\pm i\sqrt{\text{pq}}\;{\text{sech}}_{A}\left(2\xi \sqrt{-\gamma \mu }\right)\right),$$

$$F_{19}(\xi )=\sqrt{\frac{-\gamma }{\mu }}\left(-{\text{coth}}_{A}\left(2\xi \sqrt{-\gamma \mu }\right)\pm \sqrt{\text{pq}}\;{\text{csch}}_{A}\left(2\xi \sqrt{-\gamma \mu }\right)\right),$$

$$F_{20}(\xi )=-\frac{1}{2}\sqrt{\frac{-\gamma }{\mu }}\left({\text{tanh}}_{A}\left(2\xi \frac{\sqrt{-\gamma \mu }}{2}\right)+{\text{coth}}_{A}\left(2\xi \frac{\sqrt{-\gamma \mu }}{2}\right)\right).$$

**Family 5**: If β=0 and μ=γ, then


$$F_{21}(\xi )={\text{tan}}_{A}(\gamma \xi ),$$

$$F_{22}(\xi )=-{\text{cot}}_{A}(\gamma \xi ),$$

$$F_{23}(\xi )={\text{tan}}_{A}(2\gamma \xi )\pm \sqrt{\text{pq}}\;{\text{sec}}_{A}(2\gamma \xi ),$$

$$F_{24}(\xi )=-{\text{cot}}_{A}(2\gamma \xi )\pm \sqrt{\text{pq}}{\text{csc}}_{A}(2\gamma \xi ),$$

$$F_{25}(\xi )=\frac{1}{2}\left({\text{tan}}_{A}(\frac{\gamma }{2}\xi )-{\text{cot}}_{A}(\frac{\gamma }{2}\xi )\right).$$

**Family 6**: If β=0 and μ=−γ, then


$$F_{26}(\xi )=-{\text{tanh}}_{A}(\gamma \xi ),$$

$$F_{27}(\xi )=-{\text{coth}}_{A}(\gamma \xi ),$$

$$F_{28}(\xi )=-{\text{tan}}_{A}(2\gamma \xi )\pm i\sqrt{\text{pq}}\;{\text{sech}}_{A}(2\gamma \xi ),$$

$$F_{29}(\xi )=-{\text{coth}}_{A}(2\gamma \xi )\pm \sqrt{\text{pq}}{\text{csch}}_{A}(2\gamma \xi ),$$

$$F_{30}(\xi )=-\frac{1}{2}\left({\text{tanh}}_{A}(\frac{\gamma }{2}\xi )+{\text{coth}}_{A}(\frac{\gamma }{2}\xi )\right).$$

**Family 7**: If $\beta^{2}=4\gamma \mu$ ,then


$$F_{31}(\xi )=-\frac{2\gamma (\beta \xi \log(A)+2)}{\beta^{2}\xi \log(A)}.$$

**Family 8**: If β=k, γ=mk(m≠0) and μ=0, then


$$F_{32}(\xi )=A^{k\xi }-m.$$

**Family 9**: If β=μ=0, then


$$F_{33}(\xi )=\gamma \xi \log(A).$$

**Family 10**: If β=γ=0, then


$$F_{34}(\xi )=-\frac{1}{\xi \mu \log(A)}.$$

**Family 11**: If β≠0andγ=0, then


$$F_{35}(\xi )=-\frac{p\beta }{\mu \left({\text{cosh}}_{A}(\beta \xi )-{\text{sinh}}_{A}(\beta \xi )+p\right)},$$

$$F_{36}(\xi )=-\frac{q\beta }{\mu \left({\text{cosh}}_{A}(\beta \xi )-{\text{sinh}}_{A}(\beta \xi )+p\right)},$$

$$F_{37}(\xi )=\frac{\beta \left({\text{cosh}}_{A}(\beta \xi )+{\text{sinh}}_{A}(\beta \xi )\right)}{\mu \left({\text{cosh}}_{A}(\beta \xi )+{\text{sinh}}_{A}(\beta \xi )+q\right)}.$$

**Family 12**: If β=k, γ=mk(m≠0) and μ=0, then


$$F_{38}(\xi )=\frac{pA^{k\xi }}{p-mqA^{k\xi }}.$$

Where


$$sinh_{A}(\xi )=\frac{pA^{\xi }-qA^{-\xi }}{2},\;cosh_{A}(\xi )=\frac{pA^{\xi }+qA^{-\xi }}{2},\;tanh_{A}(\xi )=\frac{pA^{\xi }-qA^{-\xi }}{pA^{\xi }+qA^{-\xi }},$$

$$coth_{A}(\xi )=\frac{pA^{\xi }+qA^{-\xi }}{pA^{\xi }-qA^{-\xi }},\;sech_{A}(\xi )=\frac{2}{pA^{\xi }+qA^{-\xi }},\;csch_{A}(\xi )=\frac{2}{pA^{\xi }-qA^{-\xi }},$$

$$sin_{A}(\xi )=\frac{pA^{i\xi }-qA^{-i\xi }}{2i},\;cos_{A}(\xi )=\frac{pA^{i\xi }+qA^{-i\xi }}{2},\;tan_{A}(\xi )=-i\frac{pA^{i\xi }-qA^{-i\xi }}{pA^{i\xi }+qA^{-i\xi }},$$

$$cot_{A}(\xi )=i\frac{pA^{i\xi }+qA^{-i\xi }}{pA^{i\xi }-qA^{-i\xi }},\;sec_{A}(\xi )=\frac{2}{pA^{i\xi }+qA^{-i\xi }},\;csc_{A}(\xi )=\frac{2i}{pA^{i\xi }-qA^{-i\xi }}.$$

## 5 Extraction of solutions

Solving equations is essential for interpreting experimental or observational data, as it enables researchers to identify patterns that can guide predictions, conclusions, and theory development. This process often includes statistical analysis, curve fitting, and data modeling to enhance understanding. Where the integration constants are set to zero, balancing the nonlinear term with the second derivative results in *N* = 2.

Here are the solutions for the space-time fractional Boussinesq equation, found using the modified Sardar subequation method and the new extended direct algebraic method.

### 5.1 Modified Sardar sub-equation method

By substituting the value of *N* into Eq 9, we derive:

$$v(\xi )=a_{0}+a_{1}F(\xi )+a_{2}F(\xi {)}^{2}.$$ (13)

To move forward, we substitute Eq 13 along with its derivative into Eq 6, and then collect terms for each power of F(ξ). By setting each coefficient of F(ξ) to zero, we form a sequence of algebraic equations. Solving these equations generates several sets of solutions, each offering significant insights into the system’s dynamics and underlying structure. These computations are efficiently handled using MATHEMATICA, ensuring both accuracy and efficiency in analyzing the model.

**Set 1**

$$a_{0}=\frac{2\left(\sqrt{\beta^{2}k^{4}s_{1}^{2}s_{2}^{2}-3\delta k^{4}s_{1}^{2}s_{2}^{2}\Omega }-\beta k^{2}s_{1}s_{2}\right)}{s_{2}^{2}},\;a_{1}=0,;a_{2}=-\frac{6\delta k^{2}s_{1}}{s_{2}},\;\omega =\sqrt{bk^{2}+\frac{4k^{2}\sqrt{k^{4}s_{1}^{2}s_{2}^{2}\left(\beta^{2}-3\delta \Omega \right)}}{s_{2}}}.$$ (14)

The solutions of equation (1) are as follows:

**Family 1:** If δ≠0 , β>0 and Ω=0 , the hyperbolic solutions are,

$$u_{1}^{\pm }(x,t)=\frac{6\beta k^{2}s_{1}{\text{sech}}^{2}\left(\sqrt{\beta }\left(\frac{k{\left(\frac{1}{\Gamma (\alpha )}+x\right)}^{\alpha }}{\alpha }+\sigma +\frac{\omega {\left(\frac{1}{\Gamma (\alpha )}+t\right)}^{\alpha }}{\alpha }\right)\right)}{s_{2}}+\frac{2\left(\sqrt{\beta^{2}k^{4}s_{1}^{2}s_{2}^{2}-3\delta k^{4}s_{1}^{2}s_{2}^{2}\Omega }-\beta k^{2}s_{1}s_{2}\right)}{s_{2}^{2}}.$$ (15)

$$u_{2}^{\pm }(x,t)=\frac{2\left(\sqrt{\beta^{2}k^{4}s_{1}^{2}s_{2}^{2}-3\delta k^{4}s_{1}^{2}s_{2}^{2}\Omega }-\beta k^{2}s_{1}s_{2}\right)}{s_{2}^{2}}-\frac{6\beta k^{2}s_{1}{\text{csch}}^{2}\left(\sqrt{\beta }\left(\frac{k{\left(\frac{1}{\Gamma (\alpha )}+x\right)}^{\alpha }}{\alpha }+\sigma +\frac{\omega {\left(\frac{1}{\Gamma (\alpha )}+t\right)}^{\alpha }}{\alpha }\right)\right)}{s_{2}}.$$ (16)

**Family 2:** If $\delta =\pm E_{1}E_{2}$ , β>0 and Ω=0

$$\;\mathrm{-3pc}u_{3}^{\pm }(x,t)=+\frac{2\left(\sqrt{\beta^{2}k^{4}s_{1}^{2}s_{2}^{2}-3\delta k^{4}s_{1}^{2}s_{2}^{2}\Omega }-\beta k^{2}s_{1}s_{2}\right)}{s_{2}^{2}}\;-\frac{6e_{1}^{2}\beta \delta k^{2}s_{1}}{s_{2}\left(\left(4e_{1}^{2}-\delta \right)\cosh\left(\sqrt{\beta }\left(\frac{k{\left(\frac{1}{\Gamma (\alpha )}+x\right)}^{\alpha }}{\alpha }+\sigma +\frac{\omega {\left(\frac{1}{\Gamma (\alpha )}+t\right)}^{\alpha }}{\alpha }\right)\right)+\left(4e_{1}^{2}-\delta \right)\sinh\left(\sqrt{\beta }\left(\frac{k{\left(\frac{1}{\Gamma (\alpha )}+x\right)}^{\alpha }}{\alpha }+\sigma +\frac{\omega {\left(\frac{1}{\Gamma (\alpha )}+t\right)}^{\alpha }}{\alpha }\right)\right)\right){\;}^{2}}.$$ (17)

Where$\omega =\sqrt{bk^{2}+\frac{4k^{2}\sqrt{k^{4}s_{1}^{2}s_{2}^{2}\left(\beta^{2}-3\delta \Omega \right)}}{s_{2}}}$

**Family 3:** If δ>0 , β<0 and $\Omega =\frac{\beta^{2}}{4\delta }$

$$\;\mathrm{-6pc}u_{4}^{\pm }(x,t)=\frac{\beta k^{2}s_{1}s_{2}\left(3\tanh^{2}\left(\frac{\sqrt{-\beta }\left(\alpha \sigma +{\left(\frac{1}{\Gamma (\alpha )}+t\right)}^{\alpha }\sqrt{\frac{k^{2}\left(bs_{2}+4\sqrt{k^{4}s_{1}^{2}s_{2}^{2}\left(\beta^{2}-3\delta \Omega \right)}\right)}{s_{2}}}+k{\left(\frac{1}{\Gamma (\alpha )}+x\right)}^{\alpha }\right)}{\sqrt{2}\alpha }\right)-2\right)+2\sqrt{k^{4}s_{1}^{2}s_{2}^{2}\left(\beta^{2}-3\delta \Omega \right)}}{s_{2}^{2}}.$$ (18)

$$\;\mathrm{-6pc}u_{5}^{\pm }(x,t)=\frac{\beta k^{2}s_{1}s_{2}\left(3\coth^{2}\left(\frac{\sqrt{-\beta }\left(\alpha \sigma +{\left(\frac{1}{\Gamma (\alpha )}+t\right)}^{\alpha }\sqrt{\frac{k^{2}\left(bs_{2}+4\sqrt{k^{4}s_{1}^{2}s_{2}^{2}\left(\beta^{2}-3\delta \Omega \right)}\right)}{s_{2}}}+k{\left(\frac{1}{\Gamma (\alpha )}+x\right)}^{\alpha }\right)}{\sqrt{2}\alpha }\right)-2\right)+2\sqrt{k^{4}s_{1}^{2}s_{2}^{2}\left(\beta^{2}-3\delta \Omega \right)}}{s_{2}^{2}}.$$ (19)

$$u_{6}^{\pm }(x,t)=\frac{2\sqrt{k^{4}s_{1}^{2}s_{2}^{2}\left(\beta^{2}-3\delta \Omega \right)}+\frac{\beta k^{2}s_{1}s_{2}\left(\sinh\left(\frac{\sqrt{2}\sqrt{-\beta }\left(\alpha \sigma +k{\left(\frac{1}{\Gamma (\alpha )}+x\right)}^{\alpha }+\omega {\left(\frac{1}{\Gamma (\alpha )}+t\right)}^{\alpha }\right)}{\alpha }\right)+5i\right)}{\sinh\left(\frac{\sqrt{2}\sqrt{-\beta }\left(\alpha \sigma +k{\left(\frac{1}{\Gamma (\alpha )}+x\right)}^{\alpha }+\omega {\left(\frac{1}{\Gamma (\alpha )}+t\right)}^{\alpha }\right)}{\alpha }\right)-i}}{s_{2}^{2}}.$$ (20)

$$u_{7}^{\pm }(x,t)=\frac{2\left(\sqrt{\beta^{2}k^{4}s_{1}^{2}s_{2}^{2}-3\delta k^{4}s_{1}^{2}s_{2}^{2}\Omega }-\beta k^{2}s_{1}s_{2}\right)}{s_{2}^{2}}\;+\frac{3\beta k^{2}s_{1}{\left(\tanh\left(\frac{\sqrt{-\beta }\left(\frac{k{\left(\frac{1}{\Gamma (\alpha )}+x\right)}^{\alpha }}{\alpha }+\sigma +\frac{\omega {\left(\frac{1}{\Gamma (\alpha )}+t\right)}^{\alpha }}{\alpha }\right)}{2\sqrt{2}}\right)+i\coth\left(\frac{\sqrt{-\beta }\left(\frac{k{\left(\frac{1}{\Gamma (\alpha )}+x\right)}^{\alpha }}{\alpha }+\sigma +\frac{\omega {\left(\frac{1}{\Gamma (\alpha )}+t\right)}^{\alpha }}{\alpha }\right)}{2\sqrt{2}}\right)\right)}^{2}}{4s_{2}}.$$ (21)

$$u_{8}^{\pm }(x,t)=\frac{2\left(\sqrt{\beta^{2}k^{4}s_{1}^{2}s_{2}^{2}-3\delta k^{4}s_{1}^{2}s_{2}^{2}\Omega }-\beta k^{2}s_{1}s_{2}\right)}{s_{2}^{2}}\;+\frac{3\beta k^{2}s_{1}\left(\sqrt{W_{1}^{2}+W_{2}^{2}}-e_{1}\cosh\left(\sqrt{2}\sqrt{-\beta }\left(\frac{{\left(\frac{1}{\Gamma (\alpha )}+t\right)}^{\alpha }\sqrt{bk^{2}+\frac{4k^{2}\sqrt{k^{4}s_{1}^{2}s_{2}^{2}\left(\beta^{2}-3\delta \Omega \right)}}{s_{2}}}}{\alpha }+\frac{k{\left(\frac{1}{\Gamma (\alpha )}+x\right)}^{\alpha }}{\alpha }+\sigma \right)\right)\right){\;}^{2}}{s_{2}\left(W_{1}\sinh\left(\sqrt{2}\sqrt{-\beta }\left(\frac{{\left(\frac{1}{\Gamma (\alpha )}+t\right)}^{\alpha }\sqrt{bk^{2}+\frac{4k^{2}\sqrt{k^{4}s_{1}^{2}s_{2}^{2}\left(\beta^{2}-3\delta \Omega \right)}}{s_{2}}}}{\alpha }+\frac{k{\left(\frac{1}{\Gamma (\alpha )}+x\right)}^{\alpha }}{\alpha }+\sigma \right)\right)+W_{2}\right){\;}^{2}}.$$ (22)

$$u_{9}^{\pm }(x,t)=\frac{2\sqrt{k^{4}s_{1}^{2}s_{2}^{2}\left(\beta^{2}-3\delta \Omega \right)}+\frac{\beta k^{2}s_{1}s_{2}\left(\sinh\left(\frac{\sqrt{2}\sqrt{-\beta }\left(\alpha \sigma +k{\left(\frac{1}{\Gamma (\alpha )}+x\right)}^{\alpha }+\omega {\left(\frac{1}{\Gamma (\alpha )}+t\right)}^{\alpha }\right)}{\alpha }\right)-5i\right)}{\sinh\left(\frac{\sqrt{2}\sqrt{-\beta }\left(\alpha \sigma +k{\left(\frac{1}{\Gamma (\alpha )}+x\right)}^{\alpha }+\omega {\left(\frac{1}{\Gamma (\alpha )}+t\right)}^{\alpha }\right)}{\alpha }\right)+i}}{s_{2}^{2}}.$$ (23)

**Family 4:** If δ≠0 , β<0 and Ω=0

$$u_{10}^{\pm }(x,t)=\frac{6\beta k^{2}s_{1}\sec^{2}\left(\sqrt{-\beta }\left(\frac{{\left(\frac{1}{\Gamma (\alpha )}+t\right)}^{\alpha }\sqrt{bk^{2}+\frac{4k^{2}\sqrt{k^{4}s_{1}^{2}s_{2}^{2}\left(\beta^{2}-3\delta \Omega \right)}}{s_{2}}}}{\alpha }+\frac{k{\left(\frac{1}{\Gamma (\alpha )}+x\right)}^{\alpha }}{\alpha }+\sigma \right)\right)}{s_{2}}\;+\frac{2\left(\sqrt{\beta^{2}k^{4}s_{1}^{2}s_{2}^{2}-3\delta k^{4}s_{1}^{2}s_{2}^{2}\Omega }-\beta k^{2}s_{1}s_{2}\right)}{s_{2}^{2}}.$$ (24)

$$u_{11}^{\pm }(x,t)=\frac{6\beta k^{2}s_{1}\csc\left(\sqrt{-\beta }\left(\frac{{\left(\frac{1}{\Gamma (\alpha )}+t\right)}^{\alpha }\sqrt{bk^{2}+\frac{4k^{2}\sqrt{k^{4}s_{1}^{2}s_{2}^{2}\left(\beta^{2}-3\delta \Omega \right)}}{s_{2}}}}{\alpha }+\frac{k{\left(\frac{1}{\Gamma (\alpha )}+x\right)}^{\alpha }}{\alpha }+\sigma \right)\right){\;}^{2}}{s_{2}}\;+\frac{2\left(\sqrt{\beta^{2}k^{4}s_{1}^{2}s_{2}^{2}-3\delta k^{4}s_{1}^{2}s_{2}^{2}\Omega }-\beta k^{2}s_{1}s_{2}\right)}{s_{2}^{2}}.$$ (25)

**Family 5:** If δ>0 , β>0 and $\Omega =\frac{\beta^{2}}{4\delta }$ and $W_{1}^{2}-W_{2}^{2}>0$

$$u_{12}^{\pm }(x,t)=\frac{2\sqrt{k^{4}s_{1}^{2}s_{2}^{2}\left(\beta^{2}-3\delta \Omega \right)}-\beta k^{2}s_{1}s_{2}\left(3\tan^{2}\left(\frac{\sqrt{\beta }\left(\alpha \sigma +{\left(\frac{1}{\Gamma (\alpha )}+t\right)}^{\alpha }\sqrt{\frac{k^{2}\left(bs_{2}+4\sqrt{k^{4}s_{1}^{2}s_{2}^{2}\left(\beta^{2}-3\delta \Omega \right)}\right)}{s_{2}}}+k{\left(\frac{1}{\Gamma (\alpha )}+x\right)}^{\alpha }\right)}{\sqrt{2}\alpha }\right)+2\right)}{s_{2}^{2}}.$$ (26)

$$u_{13}^{\pm }(x,t)=\frac{2\sqrt{k^{4}s_{1}^{2}s_{2}^{2}\left(\beta^{2}-3\delta \Omega \right)}-\beta k^{2}s_{1}s_{2}\left(3\cot^{2}\left(\frac{\sqrt{\beta }\left(\alpha \sigma +k{\left(\frac{1}{\Gamma (\alpha )}+x\right)}^{\alpha }+\omega {\left(\frac{1}{\Gamma (\alpha )}+t\right)}^{\alpha }\right)}{\sqrt{2}\alpha }\right)+2\right)}{s_{2}^{2}}.$$ (27)

$$u_{14}^{\pm }(x,t)=\frac{2\left(\sqrt{\beta^{2}k^{4}s_{1}^{2}s_{2}^{2}-3\delta k^{4}s_{1}^{2}s_{2}^{2}\Omega }-\beta k^{2}s_{1}s_{2}\right)}{s_{2}^{2}}\;-\frac{3\beta k^{2}s_{1}{\left(\tan\left(\sqrt{2}\sqrt{\beta }\left(\frac{k{\left(\frac{1}{\Gamma (\alpha )}+x\right)}^{\alpha }}{\alpha }+\sigma +\frac{\omega {\left(\frac{1}{\Gamma (\alpha )}+t\right)}^{\alpha }}{\alpha }\right)\right)+\sec\left(\sqrt{2}\sqrt{\beta }\left(\frac{k{\left(\frac{1}{\Gamma (\alpha )}+x\right)}^{\alpha }}{\alpha }+\sigma +\frac{\omega {\left(\frac{1}{\Gamma (\alpha )}+t\right)}^{\alpha }}{\alpha }\right)\right)\right)}^{2}}{s_{2}}.$$ (28)

$$u_{15}^{\pm }(x,t)=\frac{2\left(\sqrt{\beta^{2}k^{4}s_{1}^{2}s_{2}^{2}-3\delta k^{4}s_{1}^{2}s_{2}^{2}\Omega }-\beta k^{2}s_{1}s_{2}\right)}{s_{2}^{2}}\;-\frac{3\beta k^{2}s_{1}{\left(\tan\left(\frac{\sqrt{\beta }\left(\frac{k{\left(\frac{1}{\Gamma (\alpha )}+x\right)}^{\alpha }}{\alpha }+\sigma +\frac{\omega {\left(\frac{1}{\Gamma (\alpha )}+t\right)}^{\alpha }}{\alpha }\right)}{2\sqrt{2}}\right)-\cot\left(\frac{\sqrt{\beta }\left(\frac{k{\left(\frac{1}{\Gamma (\alpha )}+x\right)}^{\alpha }}{\alpha }+\sigma +\frac{\omega {\left(\frac{1}{\Gamma (\alpha )}+t\right)}^{\alpha }}{\alpha }\right)}{2\sqrt{2}}\right)\right)}^{2}}{4s_{2}}.$$ (29)

$$u_{16}^{\pm }(x,t)=\frac{2\left(\sqrt{\beta^{2}k^{4}s_{1}^{2}s_{2}^{2}-3\delta k^{4}s_{1}^{2}s_{2}^{2}\Omega }-\beta k^{2}s_{1}s_{2}\right)}{s_{2}^{2}}\;-\frac{3\beta k^{2}s_{1}\left(\sqrt{W_{1}^{2}-W_{2}^{2}}-W_{1}\cos\left(\sqrt{2}\sqrt{\beta }\left(\frac{{\left(\frac{1}{\Gamma (\alpha )}+t\right)}^{\alpha }\sqrt{bk^{2}+\frac{4k^{2}\sqrt{k^{4}s_{1}^{2}s_{2}^{2}\left(\beta^{2}-3\delta \Omega \right)}}{s_{2}}}}{\alpha }+\frac{k{\left(\frac{1}{\Gamma (\alpha )}+x\right)}^{\alpha }}{\alpha }+\sigma \right)\right)\right){\;}^{2}}{s_{2}\left(W_{1}\sin\left(\sqrt{2}\sqrt{\beta }\left(\frac{{\left(\frac{1}{\Gamma (\alpha )}+t\right)}^{\alpha }\sqrt{bk^{2}+\frac{4k^{2}\sqrt{k^{4}s_{1}^{2}s_{2}^{2}\left(\beta^{2}-3\delta \Omega \right)}}{s_{2}}}}{\alpha }+\frac{k{\left(\frac{1}{\Gamma (\alpha )}+x\right)}^{\alpha }}{\alpha }+\sigma \right)\right)+W_{2}\right){\;}^{2}}.$$ (30)

$$u_{17}^{\pm }(x,t)=\frac{2\left(\sqrt{\beta^{2}k^{4}s_{1}^{2}s_{2}^{2}-3\delta k^{4}s_{1}^{2}s_{2}^{2}\Omega }-\beta k^{2}s_{1}s_{2}\right)}{s_{2}^{2}}\;-\frac{3\beta k^{2}s_{1}\cos^{2}\left(\sqrt{2}\sqrt{\beta }\left(\frac{{\left(\frac{1}{\Gamma (\alpha )}+t\right)}^{\alpha }\sqrt{bk^{2}+\frac{4k^{2}\sqrt{k^{4}s_{1}^{2}s_{2}^{2}\left(\beta^{2}-3\delta \Omega \right)}}{s_{2}}}}{\alpha }+\frac{k{\left(\frac{1}{\Gamma (\alpha )}+x\right)}^{\alpha }}{\alpha }+\sigma \right)\right)}{s_{2}\left(\sin\left(\sqrt{2}\sqrt{\beta }\left(\frac{{\left(\frac{1}{\Gamma (\alpha )}+t\right)}^{\alpha }\sqrt{bk^{2}+\frac{4k^{2}\sqrt{k^{4}s_{1}^{2}s_{2}^{2}\left(\beta^{2}-3\delta \Omega \right)}}{s_{2}}}}{\alpha }+\frac{k{\left(\frac{1}{\Gamma (\alpha )}+x\right)}^{\alpha }}{\alpha }+\sigma \right)\right)+1\right){\;}^{2}}.$$ (31)

**Family 6:** If β>0 and Ω=0

$$u_{18}^{\pm }(x,t)=-\frac{96\beta^{2}\delta k^{2}s_{1}\exp\left(2\sqrt{\beta }\left(\frac{{\left(\frac{1}{\Gamma (\alpha )}+t\right)}^{\alpha }\sqrt{bk^{2}+\frac{4k^{2}\sqrt{k^{4}s_{1}^{2}s_{2}^{2}\left(\beta^{2}-3\delta \Omega \right)}}{s_{2}}}}{\alpha }+\frac{k{\left(\frac{1}{\Gamma (\alpha )}+x\right)}^{\alpha }}{\alpha }+\sigma \right)\right)}{s_{2}{\left(\exp\left(2\sqrt{\beta }\left(\frac{{\left(\frac{1}{\Gamma (\alpha )}+t\right)}^{\alpha }\sqrt{bk^{2}+\frac{4k^{2}\sqrt{k^{4}s_{1}^{2}s_{2}^{2}\left(\beta^{2}-3\delta \Omega \right)}}{s_{2}}}}{\alpha }+\frac{k{\left(\frac{1}{\Gamma (\alpha )}+x\right)}^{\alpha }}{\alpha }+\sigma \right)\right)-4\beta \delta \right)}^{2}}\;\;+\frac{2\sqrt{\beta^{2}k^{4}s_{1}^{2}s_{2}^{2}-3\delta k^{4}s_{1}^{2}s_{2}^{2}\Omega -\beta k^{2}s_{1}s_{2}}}{s_{2}^{2}}.$$ (32)

$$u_{19}^{\pm }(x,t)=-\frac{96\beta^{2}\delta k^{2}s_{1}e^{\left(2\sqrt{\beta }\left(\frac{{\left(\frac{1}{\Gamma (\alpha )}+t\right)}^{\alpha }\sqrt{bk^{2}+\frac{4k^{2}\sqrt{k^{4}s_{1}^{2}s_{2}^{2}\left(\beta^{2}-3\delta \Omega \right)}}{s_{2}}}}{\alpha }+\frac{k{\left(\frac{1}{\Gamma (\alpha )}+x\right)}^{\alpha }}{\alpha }+\sigma \right)\right)}}{s_{2}\left(1-4\beta \delta e^{\left(2\sqrt{\beta }\left(\frac{{\left(\frac{1}{\Gamma (\alpha )}+t\right)}^{\alpha }\sqrt{bk^{2}+\frac{4k^{2}\sqrt{k^{4}s_{1}^{2}s_{2}^{2}\left(\beta^{2}-3\delta \Omega \right)}}{s_{2}}}}{\alpha }+\frac{k{\left(\frac{1}{\Gamma (\alpha )}+x\right)}^{\alpha }}{\alpha }+\sigma \right)\right)}\right){\;}^{2}}\;+\frac{2\left(\sqrt{\beta^{2}k^{4}s_{1}^{2}s_{2}^{2}-3\delta k^{4}s_{1}^{2}s_{2}^{2}\Omega }-\beta k^{2}s_{1}s_{2}\right)}{s_{2}^{2}}.$$ (33)

**Family 7:** If δ>0 and β=Ω=0,we get

$$u_{20}^{\pm }(x,t)=\frac{2\left(\sqrt{k^{4}s_{1}^{2}s_{2}^{2}\left(\beta^{2}-3\delta \Omega \right)}+k^{2}s_{1}s_{2}\left(-\beta -\frac{3\alpha^{2}}{{\left(\alpha \sigma +k{\left(\frac{1}{\Gamma (\alpha )}+x\right)}^{\alpha }+\omega {\left(\frac{1}{\Gamma (\alpha )}+t\right)}^{\alpha }\right)}^{2}}\right)\right)}{s_{2}^{2}}.$$ (34)

**Family 8:** If δ<0 and β=Ω=0,we get

$$u_{21}^{\pm }(x,t)=\frac{2\left(\sqrt{k^{4}s_{1}^{2}s_{2}^{2}\left(\beta^{2}-3\delta \Omega \right)}+k^{2}s_{1}s_{2}\left(-\beta -\frac{3\alpha^{2}}{{\left(\alpha \sigma +k{\left(\frac{1}{\Gamma (\alpha )}+x\right)}^{\alpha }+\omega {\left(\frac{1}{\Gamma (\alpha )}+t\right)}^{\alpha }\right)}^{2}}\right)\right)}{s_{2}^{2}}.$$ (35)

### 5.2 New extended direct algebraic method

By inserting the value of *N* into Eq 11, we obtain the following expression::

$$v(\xi )=a_{1}F(\xi )+a_{0}+a_{2}F(\xi {)}^{2}.$$ (36)

By substituting Eq 36 and its derivative into Eq 6 and setting the coefficients of F(ξ) to zero, we create and solve a series of algebraic equations using Mathematica, leading to multiple solutions that provide valuable insights into the system’s dynamics and structure.

$$a_{0}=-\frac{6\gamma k^{2}\mu s_{1}\log^{2}(A)}{s_{2}},\;a_{1}=-\frac{6\beta k^{2}\mu s_{1}\log^{2}(A)}{s_{2}},\;a_{2}=-\frac{6k^{2}\mu^{2}s_{1}\log^{2}(A)}{s_{2}},\;\omega =-k\sqrt{\beta^{2}k^{2}s_{1}\log^{2}(A)-4\gamma k^{2}\mu s_{1}\log^{2}(A)+b}.$$ (37)

**Family 1**: If $\beta^{2}-\gamma \mu <0$ and μ≠0, then

$$u_{22}(x,t)=\frac{3k^{2}s_{1}\log^{2}(A)\left(\beta^{2}-4\gamma \mu \right)\left({\text{tan}}_{A}\left(\frac{1}{2}\xi \sqrt{4\gamma \mu -\beta^{2}}\right){\;}^{2}+1\right)}{2s_{2}},$$ (38)

$$u_{23}(x,t)=\frac{3k^{2}s_{1}\log^{2}(A)\left(\beta^{2}-4\gamma \mu \right)\left({\text{cot}}_{A}\left(\frac{1}{2}\xi \sqrt{4\gamma \mu -\beta^{2}}\right){\;}^{2}+1\right)}{2s_{2}},$$ (39)

$$u_{24}(x,t)=-\frac{6\beta k^{2}\mu s_{1}\log^{2}(A)\left(\frac{\sqrt{4\gamma \mu -\beta^{2}}\left(\sqrt{\text{pq}}{\text{sec}}_{A}\left(\xi \sqrt{4\gamma \mu -\beta^{2}}\right)+{\text{tan}}_{A}\left(\xi \sqrt{4\gamma \mu -\beta^{2}}\right)\right)}{2\mu }-\frac{\beta }{2\mu }\right)}{s_{2}}-\frac{6\gamma k^{2}\mu s_{1}\log^{2}(A)}{s_{2}}\;-\frac{6k^{2}\mu^{2}s_{1}\log^{2}(A)\left(\frac{\sqrt{4\gamma \mu -\beta^{2}}\left(\sqrt{\text{pq}}{\text{sec}}_{A}\left(\xi \sqrt{4\gamma \mu -\beta^{2}}\right)+{\text{tan}}_{A}\left(\xi \sqrt{4\gamma \mu -\beta^{2}}\right)\right)}{2\mu }-\frac{\beta }{2\mu }\right){\;}^{2}}{s_{2}},$$ (40)

$$u_{25}(x,t)=-\frac{6\beta k^{2}\mu s_{1}\log^{2}(A)\left(\frac{\sqrt{4\gamma \mu -\beta^{2}}\left(\sqrt{\text{pq}}{\text{csc}}_{A}\left(\xi \sqrt{4\gamma \mu -\beta^{2}}\right)-{\text{cot}}_{A}\left(\xi \sqrt{4\gamma \mu -\beta^{2}}\right)\right)}{2\mu }-\frac{\beta }{2\mu }\right)}{s_{2}}-\frac{6\gamma k^{2}\mu s_{1}\log^{2}(A)}{s_{2}}\;-\frac{6k^{2}\mu^{2}s_{1}\log^{2}(A)\left(\frac{\sqrt{4\gamma \mu -\beta^{2}}\left(\sqrt{\text{pq}}{\text{csc}}_{A}\left(\xi \sqrt{4\gamma \mu -\beta^{2}}\right)-{\text{cot}}_{A}\left(\xi \sqrt{4\gamma \mu -\beta^{2}}\right)\right)}{2\mu }-\frac{\beta }{2\mu }\right){\;}^{2}}{s_{2}},$$ (41)

$$u_{26}(x,t)=-\frac{6\beta k^{2}\mu s_{1}\log^{2}(A)\left(\frac{\sqrt{4\gamma \mu -\beta^{2}}\left({\text{tan}}_{A}\left(\frac{1}{4}\xi \sqrt{4\gamma \mu -\beta^{2}}\right)-{\text{cot}}_{A}\left(\frac{1}{4}\xi \sqrt{4\gamma \mu -\beta^{2}}\right)\right)}{4\mu }-\frac{\beta }{2\mu }\right)}{s_{2}}-\frac{6\gamma k^{2}\mu s_{1}\log^{2}(A)}{s_{2}}\;-\frac{6k^{2}\mu^{2}s_{1}\log^{2}(A)\left(\frac{\sqrt{4\gamma \mu -\beta^{2}}\left({\text{tan}}_{A}\left(\frac{1}{4}\xi \sqrt{4\gamma \mu -\beta^{2}}\right)-{\text{cot}}_{A}\left(\frac{1}{4}\xi \sqrt{4\gamma \mu -\beta^{2}}\right)\right)}{4\mu }-\frac{\beta }{2\mu }\right){\;}^{2}}{s_{2}}.$$ (42)

Where $\xi =\frac{k\left(\frac{1}{\Gamma (\alpha )}+x\right){\;}^{\alpha }}{\alpha }+\frac{\omega \left(\frac{1}{\Gamma (\alpha )}+t\right){\;}^{\alpha }}{\alpha }.$

**Family 2**: If $\beta^{2}-\gamma \mu >0$ and μ≠0, then

$$u_{27}(x,t)=-\frac{3k^{2}s_{1}\log^{2}(A)\left(\beta^{2}-4\gamma \mu \right)\left({\text{tanh}}_{A}\left(\frac{1}{2}\xi \sqrt{\beta^{2}-4\gamma \mu }\right){\;}^{2}-1\right)}{2s_{2}},$$ (43)

$$u_{28}(x,t)=-\frac{3k^{2}s_{1}\log^{2}(A)\left(\beta^{2}-4\gamma \mu \right)\left({\text{coth}}_{A}\left(\frac{1}{2}\xi \sqrt{\beta^{2}-4\gamma \mu }\right){\;}^{2}-1\right)}{2s_{2}},$$ (44)

$$u_{29}(x,t)=-\frac{6\gamma k^{2}\mu s_{1}\log^{2}(A)}{s_{2}}-\frac{6\beta k^{2}\mu s_{1}\log^{2}(A)\left(-\frac{\beta }{2\mu }+\frac{\sqrt{\beta^{2}-4\gamma \mu }\left(-{\text{tanh}}_{A}\left(\xi \sqrt{\beta^{2}-4\gamma \mu }\right)+i\sqrt{\text{pq}}{\text{sech}}_{A}\left(\xi \sqrt{\beta^{2}-4\gamma \mu }\right)\right)}{2\mu }\right)}{s_{2}}\;-\frac{6k^{2}\mu^{2}s_{1}\log^{2}(A)\left(-\frac{\beta }{2\mu }+\frac{\sqrt{\beta^{2}-4\gamma \mu }\left(-{\text{tanh}}_{A}\left(\xi \sqrt{\beta^{2}-4\gamma \mu }\right)+i\sqrt{\text{pq}}{\text{sech}}_{A}\left(\xi \sqrt{\beta^{2}-4\gamma \mu }\right)\right)}{2\mu }\right){\;}^{2}}{s_{2}},$$ (45)

$$u_{30}(x,t)=-\frac{6k^{2}\mu^{2}s_{1}\log^{2}(A)\left(\frac{\sqrt{\beta^{2}-4\gamma \mu }\left(\sqrt{\text{pq}}{\text{csch}}_{A}\left(\xi \sqrt{\beta^{2}-4\gamma \mu }\right)-{\text{coth}}_{A}\left(\xi \sqrt{\beta^{2}-4\gamma \mu }\right)\right)}{2\mu }-\frac{\beta }{2\mu }\right){\;}^{2}}{s_{2}}\;-\frac{6\beta k^{2}\mu s_{1}\log^{2}(A)\left(\frac{\sqrt{\beta^{2}-4\gamma \mu }\left(\sqrt{\text{pq}}{\text{csch}}_{A}\left(\xi \sqrt{\beta^{2}-4\gamma \mu }\right)-{\text{coth}}_{A}\left(\xi \sqrt{\beta^{2}-4\gamma \mu }\right)\right)}{2\mu }-\frac{\beta }{2\mu }\right)}{s_{2}}-\frac{6\gamma k^{2}\mu s_{1}\log^{2}(A)}{s_{2}},$$ (46)

$$u_{31}(x,t)=-\frac{6k^{2}\mu^{2}s_{1}\log^{2}(A)\left(-\frac{\sqrt{\beta^{2}-4\gamma \mu }\left({\text{Tanh}}_{A}\left(\frac{1}{4}\xi \sqrt{\beta^{2}-4\gamma \mu }\right)-{\text{Coth}}_{A}\left(\frac{1}{4}\xi \sqrt{\beta^{2}-4\gamma \mu }\right)\right)}{4\mu }-\frac{\beta }{2\mu }\right){\;}^{2}}{s_{2}}\;-\frac{6\beta k^{2}\mu s_{1}\log^{2}(A)\left(-\frac{\sqrt{\beta^{2}-4\gamma \mu }\left({\text{Tanh}}_{A}\left(\frac{1}{4}\xi \sqrt{\beta^{2}-4\gamma \mu }\right)-{\text{Coth}}_{A}\left(\frac{1}{4}\xi \sqrt{\beta^{2}-4\gamma \mu }\right)\right)}{4\mu }-\frac{\beta }{2\mu }\right)}{s_{2}}-\frac{6\gamma k^{2}\mu s_{1}\log^{2}(A)}{s_{2}}.$$ (47)

Where $\xi =\frac{k\left(\frac{1}{\Gamma (\alpha )}+x\right){\;}^{\alpha }}{\alpha }+\frac{\omega \left(\frac{1}{\Gamma (\alpha )}+t\right){\;}^{\alpha }}{\alpha }.$

**Family 3**: If γμ>0 and β=0 , then

$$u_{32}(x,t)=-\frac{6k^{2}\mu s_{1}\log^{2}(A)\left(\beta \sqrt{\frac{\gamma }{\mu }}{\text{tan}}_{A}\left(\xi \sqrt{\gamma \mu }\right)+\gamma {\text{tan}}_{A}\left(\xi \sqrt{\gamma \mu }\right){\;}^{2}+\gamma \right)}{s_{2}},$$ (48)

$$u_{33}(x,t)=-\frac{6k^{2}\mu s_{1}\log^{2}(A)\left(-\beta \sqrt{\frac{\gamma }{\mu }}{\text{cot}}_{A}\left(\xi \sqrt{\gamma \mu }\right)+\gamma {\text{cot}}_{A}\left(\xi \sqrt{\gamma \mu }\right){\;}^{2}+\gamma \right)}{s_{2}},$$ (49)

$$u_{34}(x,t)=-\frac{6\beta k^{2}\mu s_{1}\log^{2}(A)\sqrt{\frac{\gamma }{\mu }}\left(\sqrt{\text{pq}}{\text{sec}}_{A}\left(2\xi \sqrt{\gamma \mu }\right)+{\text{tan}}_{A}\left(2\xi \sqrt{\gamma \mu }\right)\right)}{s_{2}}\;-\frac{6\gamma k^{2}\mu s_{1}\log^{2}(A)\left(\sqrt{\text{pq}}{\text{sec}}_{A}\left(2\xi \sqrt{\gamma \mu }\right)+{\text{tan}}_{A}\left(2\xi \sqrt{\gamma \mu }\right)\right){\;}^{2}}{s_{2}}-\frac{6\gamma k^{2}\mu s_{1}\log^{2}(A)}{s_{2}},$$ (50)

$$u_{35}(x,t)=-\frac{6\beta k^{2}\mu s_{1}\log^{2}(A)\sqrt{\frac{\gamma }{\mu }}\left(\sqrt{\text{pq}}{\text{csc}}_{A}\left(2\xi \sqrt{\gamma \mu }\right)-{\text{cot}}_{A}\left(2\xi \sqrt{\gamma \mu }\right)\right)}{s_{2}}\;-\frac{6\gamma k^{2}\mu s_{1}\log^{2}(A)\left(\sqrt{\text{pq}}{\text{csc}}_{A}\left(2\xi \sqrt{\gamma \mu }\right)-{\text{cot}}_{A}\left(2\xi \sqrt{\gamma \mu }\right)\right){\;}^{2}}{s_{2}}-\frac{6\gamma k^{2}\mu s_{1}\log^{2}(A)}{s_{2}},$$ (51)

$$u_{36}(x,t)=-\frac{3\beta k^{2}\mu s_{1}\log^{2}(A)\sqrt{\frac{\gamma }{\mu }}\left({\text{tan}}_{A}\left(\frac{1}{2}\xi \sqrt{\gamma \mu }\right)-{\text{cot}}_{A}\left(\frac{1}{2}\xi \sqrt{\gamma \mu }\right)\right)}{s_{2}}\;-\frac{3\gamma k^{2}\mu s_{1}\log^{2}(A)\left({\text{tan}}_{A}\left(\frac{1}{2}\xi \sqrt{\gamma \mu }\right)-{\text{cot}}_{A}\left(\frac{1}{2}\xi \sqrt{\gamma \mu }\right)\right){\;}^{2}}{2s_{2}}-\frac{6\gamma k^{2}\mu s_{1}\log^{2}(A)}{s_{2}}.$$ (52)

Where $\xi =\frac{k\left(\frac{1}{\Gamma (\alpha )}+x\right){\;}^{\alpha }}{\alpha }+\frac{\omega \left(\frac{1}{\Gamma (\alpha )}+t\right){\;}^{\alpha }}{\alpha }.$

**Family 4**: If γμ<0 and β=0 , then

$$u_{37}(x,t)=\frac{6k^{2}\mu s_{1}\log^{2}(A)\left(\beta \sqrt{-\frac{\gamma }{\mu }}{\text{tanh}}_{A}\left(\xi \sqrt{-\gamma \mu }\right)+\gamma {\text{tanh}}_{A}\left(\xi \sqrt{-\gamma \mu }\right){\;}^{2}-\gamma \right)}{s_{2}},$$ (53)

$$u_{38}(x,t)=-\frac{6k^{2}\mu s_{1}\log^{2}(A)\left(-\beta {\text{cot}}_{A}(\gamma \xi )+\mu {\text{cot}}_{A}(\gamma \xi ){\;}^{2}+\gamma \right)}{s_{2}},$$ (54)

$$u_{39}(x,t)=-\frac{6\gamma k^{2}\mu s_{1}\log^{2}(A)}{s_{2}}-\frac{6\beta k^{2}\mu s_{1}\log^{2}(A)\sqrt{-\frac{\gamma }{\mu }}\left(-{\text{tanh}}_{A}\left(2\xi \sqrt{-\gamma \mu }\right)+i\sqrt{\text{pq}}{\text{sech}}_{A}\left(2\xi \sqrt{-\gamma \mu }\right)\right)}{s_{2}}\;+\frac{6\gamma k^{2}\mu s_{1}\log^{2}(A)\left(-{\text{Tanh}}_{A}\left(2\xi \sqrt{-\gamma \mu }\right)+i\sqrt{\text{pq}}{\text{Sech}}_{A}\left(2\xi \sqrt{-\gamma \mu }\right)\right){\;}^{2}}{s_{2}},$$ (55)

$$u_{40}(x,t)=-\frac{6\gamma k^{2}\mu s_{1}\log^{2}(A)}{s_{2}}-\frac{6\beta k^{2}\mu s_{1}\log^{2}(A)\sqrt{-\frac{\gamma }{\mu }}\left(\sqrt{\text{pq}}{\text{csch}}_{A}\left(2\xi \sqrt{-\gamma \mu }\right)-{\text{coth}}_{A}\left(2\xi \sqrt{-\gamma \mu }\right)\right)}{s_{2}}\;+\frac{6\gamma k^{2}\mu s_{1}\log^{2}(A)\left(\sqrt{\text{pq}}{\text{csch}}_{A}\left(2\xi \sqrt{-\gamma \mu }\right)-{\text{coth}}_{A}\left(2\xi \sqrt{-\gamma \mu }\right)\right){\;}^{2}}{s_{2}},$$ (56)

$$u_{41}(x,t)=\frac{3\beta k^{2}\mu s_{1}\log^{2}(A)\sqrt{-\frac{\gamma }{\mu }}\left({\text{coth}}_{A}\left(\frac{1}{2}\xi \sqrt{-\gamma \mu }\right)+{\text{tanh}}_{A}\left(\frac{1}{2}\xi \sqrt{-\gamma \mu }\right)\right)}{s_{2}}\;+\frac{3\gamma k^{2}\mu s_{1}\log^{2}(A)\left({\text{coth}}_{A}\left(\frac{1}{2}\xi \sqrt{-\gamma \mu }\right)+{\text{tanh}}_{A}\left(\frac{1}{2}\xi \sqrt{-\gamma \mu }\right)\right){\;}^{2}}{2s_{2}}-\frac{6\gamma k^{2}\mu s_{1}\log^{2}(A)}{s_{2}}.$$ (57)

Where $\xi =\frac{k\left(\frac{1}{\Gamma (\alpha )}+x\right){\;}^{\alpha }}{\alpha }+\frac{\omega \left(\frac{1}{\Gamma (\alpha )}+t\right){\;}^{\alpha }}{\alpha }.$

**Family 5**: If β=0 and μ=γ, then

$$u_{42}(x,t)=-\frac{6k^{2}\mu s_{1}\log^{2}(A)\left(\beta {\text{tan}}_{A}(\gamma \xi )+\mu {\text{tan}}_{A}(\gamma \xi ){\;}^{2}+\gamma \right)}{s_{2}},$$ (58)

$$u_{43}(x,t)=-\frac{6k^{2}\mu s_{1}\log^{2}(A)\left(-\beta {\text{cot}}_{A}(\gamma \xi )+\mu {\text{cot}}_{A}(\gamma \xi ){\;}^{2}+\gamma \right)}{s_{2}},$$ (59)

$$u_{44}(x,t)=-\frac{6k^{2}\mu s_{1}\log^{2}(A)\left(\mu \text{pq}{\text{sec}}_{A}(2\gamma \xi ){\;}^{2}+\sqrt{\text{pq}}{\text{sec}}_{A}(2\gamma \xi )\left(2\mu {\text{tan}}_{A}(2\gamma \xi )+\beta \right)+\beta {\text{tan}}_{A}(2\gamma \xi )+\mu {\text{Tan}}_{A}(2\gamma \xi ){\;}^{2}+\gamma \right)}{s_{2}},$$ (60)

$$u_{45}(x,t)=-\frac{6\beta k^{2}\mu s_{1}\log^{2}(A)\left(\sqrt{\text{pq}}{\text{csc}}_{A}(2\gamma \xi )-{\text{cot}}_{A}(2\gamma \xi )\right)}{s_{2}}\;-\frac{6k^{2}\mu^{2}s_{1}\log^{2}(A)\left(\sqrt{\text{pq}}{\text{csc}}_{A}(2\gamma \xi )-{\text{cot}}_{A}(2\gamma \xi )\right){\;}^{2}}{s_{2}}-\frac{6\gamma k^{2}\mu s_{1}\log^{2}(A)}{s_{2}},$$ (61)

$$u_{46}(x,t)=-\frac{3\beta k^{2}\mu s_{1}\log^{2}(A)\left({\text{tan}}_{A}\left(\frac{\gamma \xi }{2}\right)-{\text{cot}}_{A}\left(\frac{\gamma \xi }{2}\right)\right)}{s_{2}}\;-\frac{3k^{2}\mu^{2}s_{1}\log^{2}(A)\left({\text{tan}}_{A}\left(\frac{\gamma \xi }{2}\right)-{\text{cot}}_{A}\left(\frac{\gamma \xi }{2}\right)\right){\;}^{2}}{2s_{2}}-\frac{6\gamma k^{2}\mu s_{1}\log^{2}(A)}{s_{2}}.$$ (62)

Where $\xi =\frac{k\left(\frac{1}{\Gamma (\alpha )}+x\right){\;}^{\alpha }}{\alpha }+\frac{\omega \left(\frac{1}{\Gamma (\alpha )}+t\right){\;}^{\alpha }}{\alpha }.$

**Family 6**: If β=0 and μ=−γ, then

$$u_{47}(x,t)=-\frac{6k^{2}\mu s_{1}\log^{2}(A)\left(-\beta {\text{tanh}}_{A}(\gamma \xi )+\mu {\text{tanh}}_{A}(\gamma \xi ){\;}^{2}+\gamma \right)}{s_{2}},$$ (63)

$$u_{48}(x,t)=-\frac{6k^{2}\mu s_{1}\log^{2}(A)\left(-\beta {\text{coth}}_{A}(\gamma \xi )+\mu {\text{coth}}_{A}(\gamma \xi ){\;}^{2}+\gamma \right)}{s_{2}},$$ (64)

$$u_{49}(x,t)=-\frac{6\beta k^{2}\mu s_{1}\log^{2}(A)\left(-{\text{tanh}}_{A}(2\gamma \xi )+i\sqrt{\text{pq}}{\text{sech}}_{A}(2\gamma \xi )\right)}{s_{2}}\;-\frac{6k^{2}\mu^{2}s_{1}\log^{2}(A)\left(-{\text{tanh}}_{A}(2\gamma \xi )+i\sqrt{\text{pq}}{\text{sech}}_{A}(2\gamma \xi )\right){\;}^{2}}{s_{2}}-\frac{6\gamma k^{2}\mu s_{1}\log^{2}(A)}{s_{2}},$$ (65)

$$u_{50}(x,t)=-\frac{6\beta k^{2}\mu s_{1}\log^{2}(A)\left(\sqrt{pq}{\text{csch}}_{A}(2\gamma \xi )-{\text{coth}}_{A}(2\gamma \xi )\right)}{s_{2}}\;-\frac{6k^{2}\mu^{2}s_{1}\log^{2}(A)\left(\sqrt{pq}{\text{csch}}_{A}(2\gamma \xi )-{\text{coth}}_{A}(2\gamma \xi )\right){\;}^{2}}{s_{2}}-\frac{6\gamma k^{2}\mu s_{1}\log^{2}(A)}{s_{2}},$$ (66)

$$u_{51}(x,t)=-\frac{3\beta k^{2}\mu s_{1}\log^{2}(A)\left(-{\text{coth}}_{A}\left(\frac{\gamma \xi }{2}\right)-{\text{tanh}}_{A}\left(\frac{\gamma \xi }{2}\right)\right)}{s_{2}}\;-\frac{3k^{2}\mu^{2}s_{1}\log^{2}(A)\left(-{\text{coth}}_{A}\left(\frac{\gamma \xi }{2}\right)-{\text{tanh}}_{A}\left(\frac{\gamma \xi }{2}\right)\right){\;}^{2}}{2s_{2}}-\frac{6\gamma k^{2}\mu s_{1}\log^{2}(A)}{s_{2}}.$$ (67)

Where $\xi =\frac{k\left(\frac{1}{\Gamma (\alpha )}+x\right){\;}^{\alpha }}{\alpha }+\frac{\omega \left(\frac{1}{\Gamma (\alpha )}+t\right){\;}^{\alpha }}{\alpha }.$

**Family 7**: If $\beta^{2}=4\gamma \mu$ ,then

$$u_{52}(x,t)=\frac{6\gamma k^{2}\mu s_{1}\left(\beta^{2}\xi^{2}\log^{2}(A)\left(\beta^{2}-4\gamma \mu \right)+4\beta \xi \log(A)\left(\beta^{2}-4\gamma \mu \right)-16\gamma \mu \right)}{\beta^{4}\xi^{2}s_{2}}.$$ (68)

**Family 8**: If β=k, γ=mk(m≠0) and μ=0, then

$$u_{53}(x,t)=-\frac{6k^{2}\mu s_{1}\log^{2}(A)\left(\mu A^{2\#x2009;L\xi }+A^{L\xi }(\beta -2\mu m)+\gamma +\mu m^{2}-\beta m\right)}{s_{2}}.$$ (69)

**Family 9**: If β=μ=0, then

$$u_{54}(x,t)=\frac{6\gamma^{2}k^{2}\mu^{2}\xi s_{1}\log^{4}(A)\left(6\beta k^{2}s_{1}\log(A)-\xi s_{2}\right)}{s_{2}^{2}}.$$ (70)

**Family 10**: If β=γ=0, then

$$u_{55}(x,t)=-\frac{6k^{2}\mu s_{1}\log(A)\left(\gamma \xi^{3}s_{2}\sigma^{3}\log(A)+6\beta k^{2}\mu^{2}s_{1}\right)}{\xi^{3}\sigma^{3}s_{2}^{2}}.$$ (71)

Where $\xi =\frac{k\left(\frac{1}{\Gamma (\alpha )}+x\right){\;}^{\alpha }}{\alpha }+\frac{\omega \left(\frac{1}{\Gamma (\alpha )}+t\right){\;}^{\alpha }}{\alpha }.$

**Family 11**: If β≠0andγ=0, then

$$u_{56}(x,t)=\frac{6k^{2}s_{1}\log^{2}(A)\left(-\frac{\beta^{2}p\left({\text{cosh}}_{A}(\beta \xi )-{\text{sinh}}_{A}(\beta \xi )+2p\right)}{\left({\text{cosh}}_{A}(\beta \xi )-{\text{sinh}}_{A}(\beta \xi )+p\right){\;}^{2}}-\gamma \mu \right)}{s_{2}}.$$ (72)

$$u_{57}(x,t)=\frac{6k^{2}s_{1}\log^{2}(A)\left(-\frac{\beta^{2}q\left({\text{cosh}}_{A}(\beta \xi )-{\text{sinh}}_{A}(\beta \xi )+p+q\right)}{\left({\text{cosh}}_{A}(\beta \xi )-{\text{sinh}}_{A}(\beta \xi )+p\right){\;}^{2}}-\gamma \mu \right)}{s_{2}}.$$ (73)

$$u_{58}(x,t)=\frac{6k^{2}s_{1}\log^{2}(A)\left(-\frac{\beta^{2}\left({\text{cosh}}_{A}(\beta \xi )+{\text{sinh}}_{A}(\beta \xi )\right)}{{\text{cosh}}_{A}(\beta \xi )+{\text{sinh}}_{A}(\beta \xi )+q}-\frac{\beta^{2}\left({\text{cosh}}_{A}(\beta \xi )+{\text{sinh}}_{A}(\beta \xi )\right){\;}^{2}}{\left({\text{cosh}}_{A}(\beta \xi )+{\text{sinh}}_{A}(\beta \xi )+q\right){\;}^{2}}-\gamma \mu \right)}{s_{2}}.$$ (74)

Where $\xi =\frac{k\left(\frac{1}{\Gamma (\alpha )}+x\right){\;}^{\alpha }}{\alpha }+\frac{\omega \left(\frac{1}{\Gamma (\alpha )}+t\right){\;}^{\alpha }}{\alpha }.$

**Family 12**: If β=k, γ=mk(m≠0) and μ=0, then

$$u_{59}(x,t)=-\frac{6k^{2}\mu s_{1}\log^{2}(A)\left(A^{2\#x2009;L\xi }\left(\gamma m^{2}q^{2}-\beta mpq+\mu p^{2}\right)+pA^{L\xi }(\beta p-2\gamma mq)+\gamma p^{2}\right)}{s_{2}{\left(p-mqA^{L\xi }\right)}^{2}}.$$ (75)

## 6 Graphical representations

This section presents graphical representations of selected solutions derived from the study. The obtained solutions reveal a variety of wave patterns, which have valuable applications in nonlinear evolution equations. Graphical illustrations, including 2D and 3D plots for various values of *α*, are utilized to clearly depict these diverse wave structures, making complex behaviors easier to interpret. Graphs serve as a vital component for both explaining phenomena and illustrating specific cases. The primary results of this work involve traveling wave solutions for the space-time fractional Boussinesq equation, achieved using the modified Sardar sub-equation method and the new extended direct algebraic method. This research provides several solutions expressed as trigonometric, hyperbolic, complex, and rational functions. By adjusting equation parameters, a diverse set of graphs has been generated, each offering insights into solution behaviors and distinct characteristics. The inclusion of negative time also allows exploration of preceding conditions, further enhancing the analysis of wave dynamics.The following provides a comparison of selected solutions obtained from both techniques.

**Fig 1:**
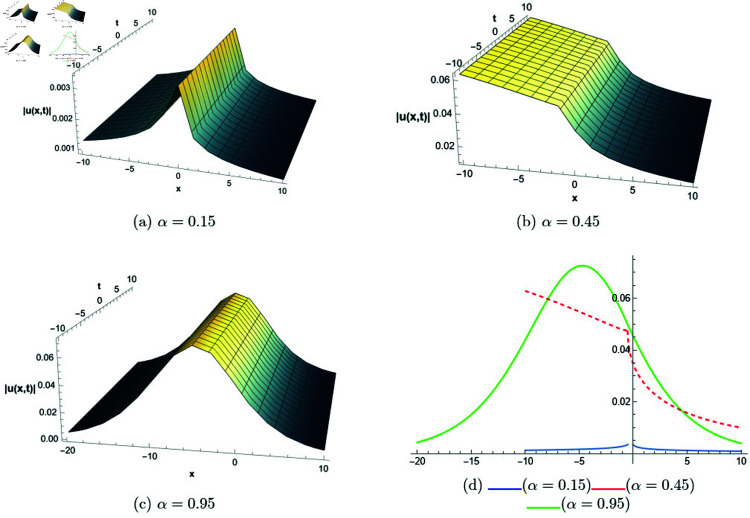
The comparative profile of solution *u*_1_(*x*,*t*) for different values of *α* using 3D and 2D plots.

**Fig 2:**
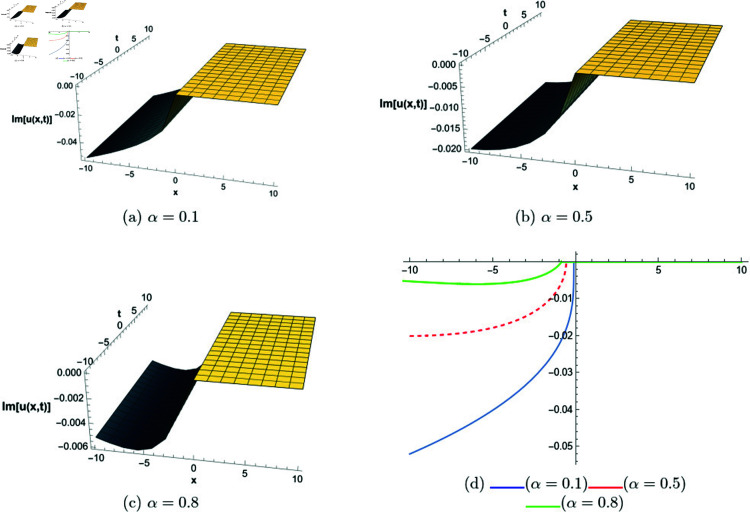
The comparative profile of solution *u*_2_(*x*,*t*) for different values of *α* using 3D and 2D plots.

**Fig 3:**
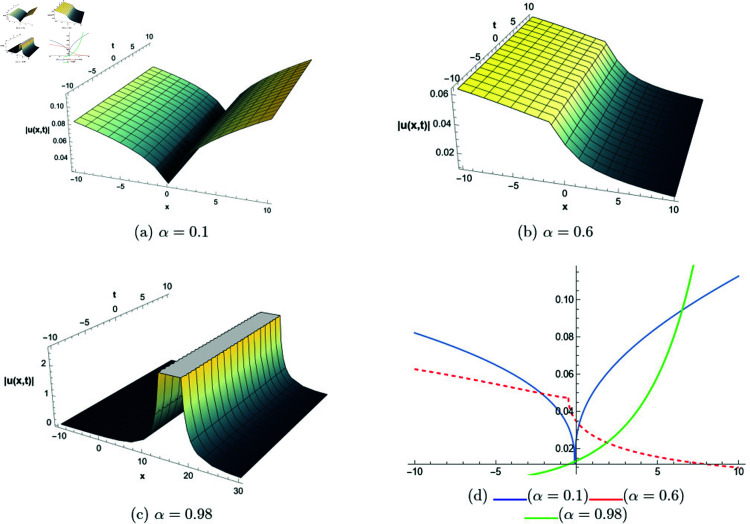
The comparative profile of solution *u*_5_(*x*,*t*) for different values of *α* using 3D and 2D plots.

**Fig 4:**
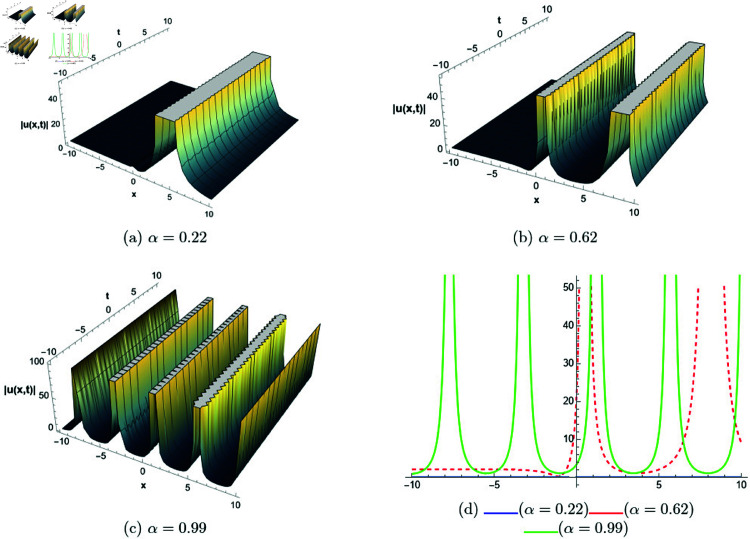
The comparative profile of solution *u*_12_(*x*,*t*) for different values of *α* using 3D and 2D plots.

**Fig 5:**
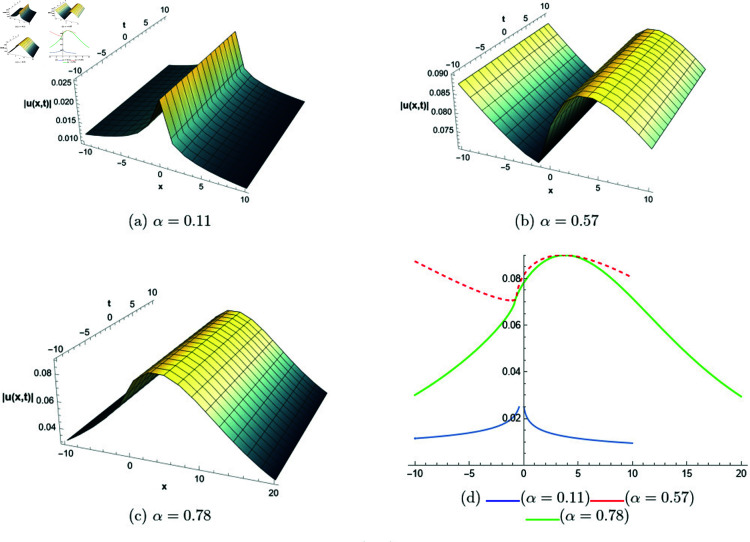
The comparative profile of solution *u*_18_(*x*,*t*) for different values of *α* using 3D and 2D plots.

**Fig 6:**
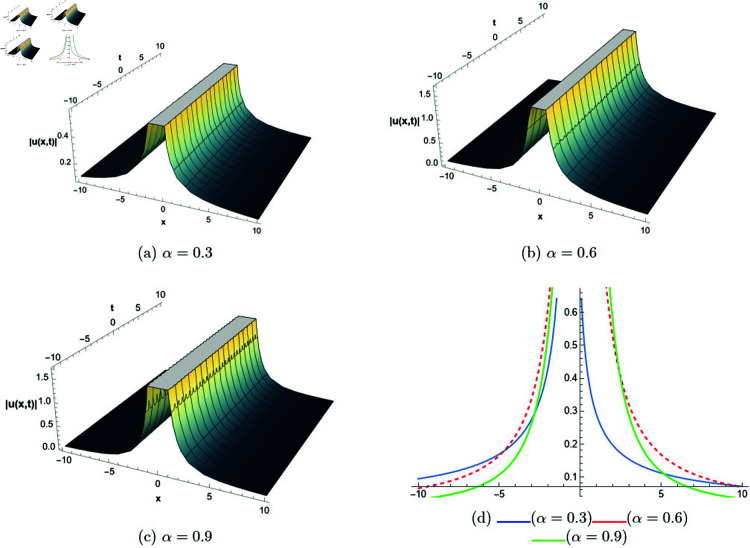
The comparative profile of solution *u*_20_(*x*,*t*) for different values of *α* using 3D and 2D plots.

**Modified Sardar sub-equation method:** Figs 1 to 6 show solutions by modified Sardar sub-equation method. Fig 1 presents the bright and anti-kink type solitons for the solution *u*_1_(*x*,*t*), depicted for varying values of *α*. The plots are generated with specific parameters: β=0.6, δ=0.5, Ω=0, σ=0.3, *s*_1_ = 0.4, *s*_2_ = 0.8, *k* = 0.2, and *b* = 1, within the intervals x∈[−20,10] and t∈[−10,10]. This figure allows us to observe the influence of different fractional orders on the soliton profiles, illustrating how changes in *α* modify their shapes and characteristics. In Fig 2, kink-type solitons for the solution *u*_2_(*x*,*t*) are displayed for various values of *α*. These plots are created using the following parameter settings: β=1, δ=1, Ω=0, σ=1, *s*_1_ = 1, *s*_2_ = 1, *k* = −0.1, and *b* = 1, over the ranges x∈[−10,10] and t∈[−10,10]. This figure suggests that changes in the fractional order *α* do not alter the overall form of the soliton, indicating stability in its shape across different fractional values. Fig 3 presents dark, anti-kink, and bright singular solitons for the solution *u*_5_(*x*,*t*), shown for different values of *α*. The plots are generated with parameters set to: β=−1, δ=1, Ω=0.25, σ=3, *s*_1_ = −2, *s*_2_ = −1, *k* = −0.2, and *b* = 0.5, within the intervals x∈[−10,10] and t∈[−10,10]. The figure illustrates how varying *α* results in noticeable shifts in the soliton types, showcasing the influence of the fractional order on soliton characteristics. In Fig 4, bright singular and periodic singular solitons for the solution *u*_12_(*x*,*t*) are displayed across different values of *α*. The plots are generated using the following parameter values: β=1, δ=1, Ω=0.25, σ=1, *s*_1_ = 1, *s*_2_ = 1, *k* = 1, and *b* = 1, over the ranges x∈[−10,10] and t∈[−10,10]. This figure highlights the noticeable changes in soliton types as *α* varies, demonstrating the effect of the fractional order on the characteristics of the solitons. Fig 5 showcases bright, dark, and bell-shaped solitons for the solution *u*_18_(*x*,*t*) across various values of *α*. The plots are generated using parameter values of β=1.5, δ=−1, Ω=0, σ=0.1, *s*_1_ = 0.1, *s*_2_ = 0.1, *k* = 0.1, and *b* = 1, within the intervals x∈[−10,10] and t∈[−10,10]. The figure reveals significant changes in the soliton forms as *α* varies, illustrating the strong impact of fractional order adjustments on soliton characteristics. In Fig 6, bright singular solitons for the solution *u*_20_(*x*,*t*) are depicted for a range of *α* values. The soliton profiles are plotted with the parameters set to β=0, δ=1, Ω=0, σ=1, *s*_1_ = 0.4, *s*_2_ = −1, *k* = −1, and *b* = −0.1, covering the intervals x∈[−10,10] and t∈[−10,10]. This figure provides a visual comparison of the soliton structures as influenced by changes in *α*.

**Fig 7:**
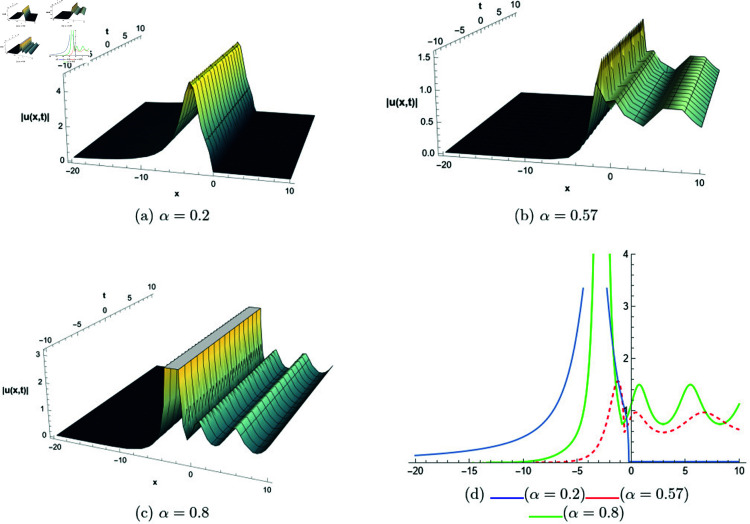
The comparative profile of solution *u*_22_(*x*,*t*) for different values of *α* using 3D and 2D plots.

**Fig 8:**
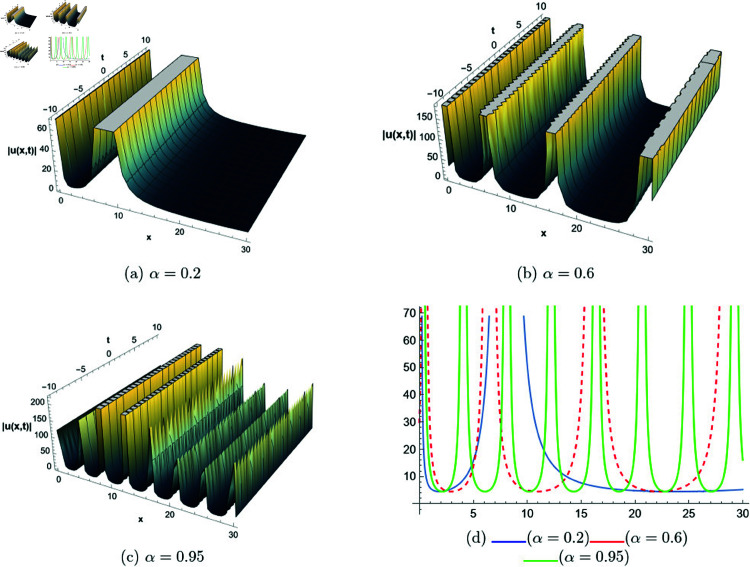
The comparative profile of solution *u*_29_(*x*,*t*) for different values of *α* using 3D and 2D plots.

**Fig 9:**
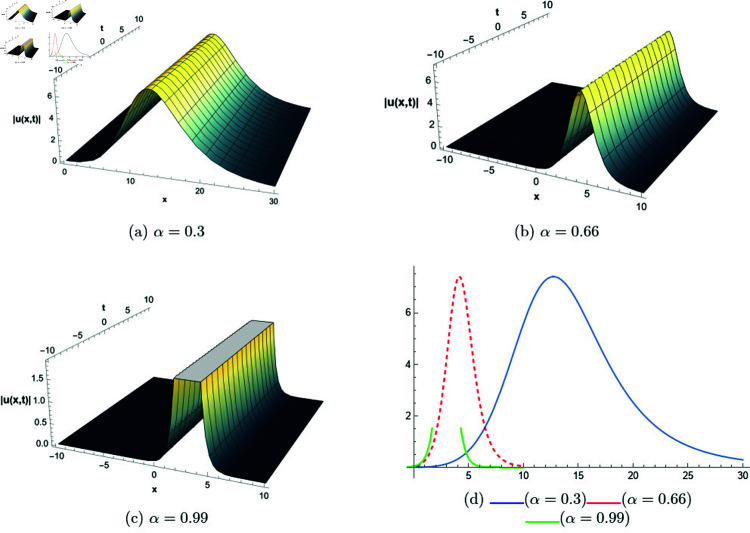
The comparative profile of solution *u*_29_(*x*,*t*) for different values of *α* using 3D and 2D plots.

**Fig 10:**
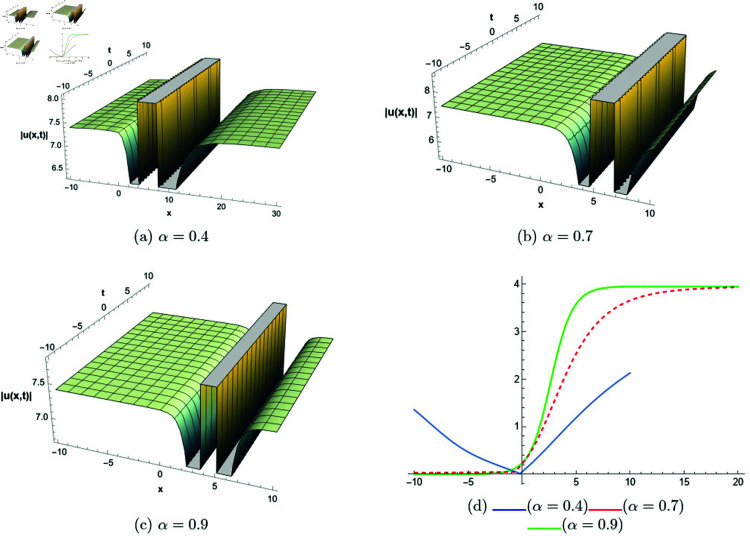
The comparative profile of solution *u*_31_(*x*,*t*) for different values of *α* using 3D and 2D plots.

**Fig 11:**
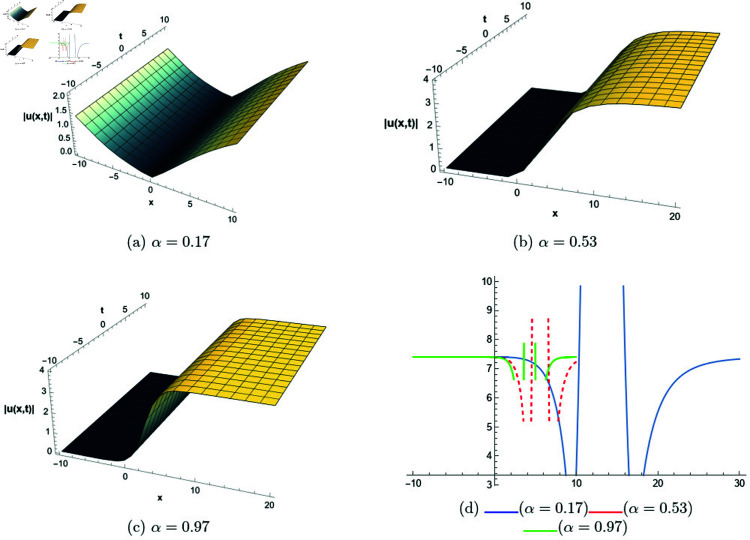
The comparative profile of solution *u*_56_(*x*,*t*) for different values of *α* using 3D and 2D plots.

**New extended direct algebraic method:** Fig 7 illustrates bright and kink-type solitons for the solution *u*_22_(*x*,*t*) across various values of *α*. The profiles are plotted using parameters: β=1, μ=1, γ=1, *k* = 1, *s*_1_ = 0.5, *s*_2_ = 1.5, *b* = 1, *p* = 1, *q* = 2, and *A* = 2.7, with *x* ranging from −20 to 10. This figure visually demonstrates how soliton structures vary with different values of *α*, allowing for a comparative analysis of their form and characteristics. In Fig 8, periodic singular solitons for the solution *u*_25_(*x*,*t*) are displayed across different values of *α*. These profiles are generated with the following parameter values: β=1, μ=1, γ=1, *k* = 1, *s*_1_ = −1, *s*_2_ = 1, *b* = 1, *p* = −1, *q* = 1, and *A* = 2.7, covering the range x∈[0,30]. Fig 9 presents various types of bell-shaped solitons for the solution *u*_29_(*x*,*t*), shown for a range of *α* values. The soliton profiles are generated using parameters: β=3, μ=1, γ=1, *k* = 1, *s*_1_ = 1, *s*_2_ = 1, *b* = −1, *p* = −1, *q* = 1, and *A* = 2.7, within the interval x∈[0,30]. This figure provides insight into the behavior and structural differences of bell solitons as influenced by varying *α*. Fig (10) displays dark, bright, and singular solitons for the solution *u*_31_(*x*,*t*) across a range of α values. The soliton profiles are plotted with parameters: β=3, μ=1, γ=1, *k* = 1, *s*_1_ = 1, *s*_2_ = 1, *b* = −1, *p* = −1, *q* = 0.5, and *A* = 2.7, covering *x* within the range [−10,10]. This figure illustrates the variations in soliton structure and behavior resulting from changes in *α*, highlighting the distinctive characteristics of each soliton type. Fig 11 presents the profiles of dark and kink solitons for the solution *u*_56_(*x*,*t*), showcasing how these solitons evolve over a range of *α* values. The parameters used in the plots are β=1, μ=1, γ=0, *k* = 1, *s*_1_ = 0.5, *s*_2_ = 1.5, *b* = 1, *p* = 1, *q* = 2, and *A* = 2.7, with *x* spanning from −10 to 20. The parameters *k* and *ω* play a crucial role in defining the soliton dynamics, where *k* represents the coefficient of the spatial variable and *ω* determines the speed of the traveling wave. Both parameters hold significant physical importance, as *ω* is inherently dependent on *k* and *s*_1_. Even slight variations in these parameters can drastically alter the soliton’s behavior, leading to diverse wave structures. The distinct values of *k* and *s*_1_ used across all figures further illustrate this diversity, emphasizing the sensitivity of the model to parameter adjustments and showcasing the rich dynamical characteristics of the soliton solutions.

Dark solitons are wave solutions that appear as localized dips in amplitude, forming intensity minima against a continuous wave background. These solitons maintain their shape as they propagate, making them important in nonlinear wave dynamics. In contrast, bright solitons exhibit peaks in amplitude, creating intensity maxima against a zero or low-intensity background. These solutions arise due to the balance between dispersion and nonlinearity, allowing them to travel without distortion. Singular periodic solitons are another class of wave solutions that display periodic oscillations but contain singularities such as sharp peaks or undefined points within each period. These solitons demonstrate complex behavior, making them significant in the study of nonlinear wave equations. Kink solitons are known for their step-like transition between two distinct states, maintaining a stable shape as they travel. Their counterparts, anti-kink solitons, transition in the opposite direction but share similar stability properties. These solitons are widely studied in various physical systems, including fluid dynamics and optical fibers. Bell-shaped solitons, on the other hand, have a smooth, symmetric peak resembling the shape of a bell curve. They are commonly observed in nonlinear wave models and describe localized wave structures with minimal dispersion. Each of these soliton types plays a crucial role in understanding wave propagation and nonlinear dynamics in different scientific fields.

## 7 Phase plane analysis

Phase plane analysis is a mathematical technique used to study the behavior of dynamical systems by plotting the system’s state variables, typically position and velocity, on a two-dimensional plane. It helps visualize the system’s trajectories, equilibrium points, and stability, offering insights into phenomena like oscillations, bifurcations, and chaotic behavior. By analyzing the phase plane, researchers can identify the long-term behavior of the system, such as periodic motion or convergence to steady states, and understand the system’s response to different initial conditions or parameters. This method is widely used in physics, engineering, and other fields to explore nonlinear systems [37, 38].

### 7.1 Bifurcation analysis

Bifurcation analysis studies changes in a system’s behavior as a key parameter varies, identifying points where small shifts cause transitions, like from stability to instability or periodic to chaotic motion. It helps predict critical transitions and map stability regions, making it valuable in physics, biology, and engineering [39, 40].

In this section, we apply bifurcation theory to analyze Eq 6. By implementing a Galilean transformation, we reformulate Eq 6 into a two-dimensional dynamical system, represented as:

$$\frac{du}{d\xi }=L\;\frac{dL}{d\xi }=N_{1}u-N_{2}u^{2}=T$$ (76)

Here, $N_{1}=\frac{\omega^{2}-bk^{2}}{k^{2}s_{1}}$ and $N_{2}=\frac{s_{2}}{k^{2}s_{1}}$. Next, we will examine the phase portraits associated with the bifurcations in system (76), concentrating on the parameter space defined by *N*_1_ and *N*_2_. This analysis aims to reveal how variations in these parameters influence the system’s dynamic behavior, shedding light on the stability and transitions of equilibrium points and other dynamic characteristics. This system exhibits two equilibrium points.


$$G_{1}=(0,0)\;\;G_{2}=(\frac{N_{1}}{N_{2}},0)$$

The Jacobian matrix of above system is


$$J(u,L)=\det(\begin{array}{cc} 0 & 1\\ N_{1}-2N_{2}u & 0 \end{array})=-N_{1}+2N_{2}u$$

Therefore, the point (u,L) acts as a saddle if J(u,L)<0, indicating instability. It behaves as a center when J(u,L)>0, showing neutral stability and periodic behavior. When J(u,L)=0, the point becomes a cusp, marking a critical transition in the system’s dynamics.

• **Case 1:** Let *N*_1_>0 , *N*_2_>0.

**Fig 12:**
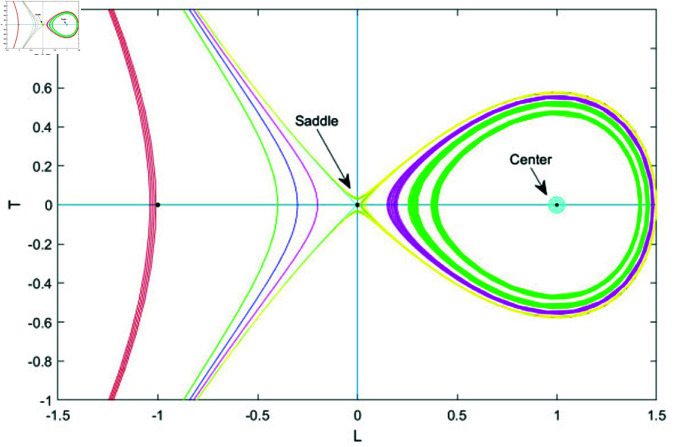
Graphical visualization of case 1 under the parametric value k= 1, b=1 and ω=2, *s*_1_ = 3.

In case 1, system (76) has two equilibrium points: *G*_1_ = (0,0) and *G*_2_ = (1,0). Here, *G*_1_ functions as a saddle point, while *G*_2_ serves as a center point. To gain further insights into the system’s dynamics, we analyze how varying *N*_1_ and *N*_2_ affects behavior by choosing different parameter values. The resulting phase plots are illustrated in Fig (12).

• **Case 2:** Let *N*_1_>0 and *N*_2_<0.

**Fig 13:**
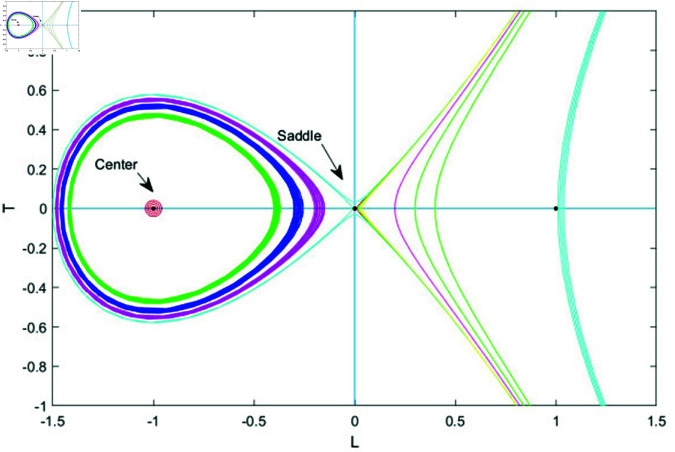
Graphical visualization of case 2 under the parametric value k= 1, b=1 and ω=2, *s*_1_ = 3.

In case 2, system (76) has two equilibrium points: *G*_1_ = (0,0) and *G*_2_ = (−1,0). Here, *G*_1_ acts as a saddle point, while *G*_2_ functions as a center point. The phase plots corresponding to this case are displayed in Fig (13).

• **Case 3:** Let *N*_1_<0 and *N*_2_>0.

**Fig 14:**
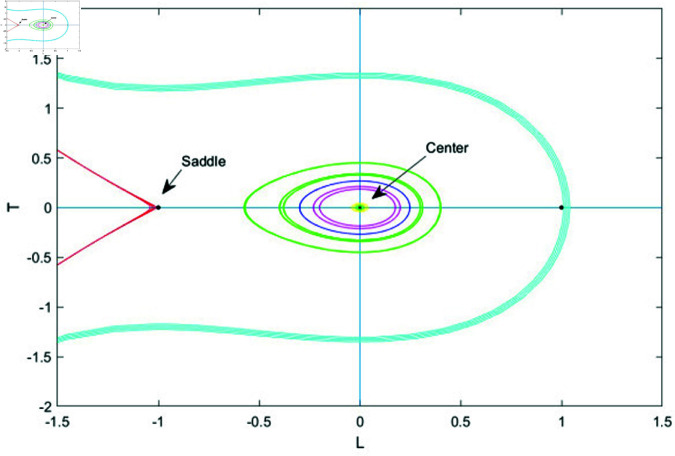
Graphical visualization of case 3 under the parametric value k= 1, b=1 and ω=2, *s*_1_ = 3.

In case 3, system (76) has two equilibrium points: *G*_1_ = (0,0) and *G*_2_ = (−1,0). In this scenario, *G*_1_ functions as the center point, while *G*_2_ acts as the saddle point. The phase plots illustrating this case are shown in Fig 14.

• **Case 4:** Let *N*_1_<0 and *N*_2_<0.

**Fig 15:**
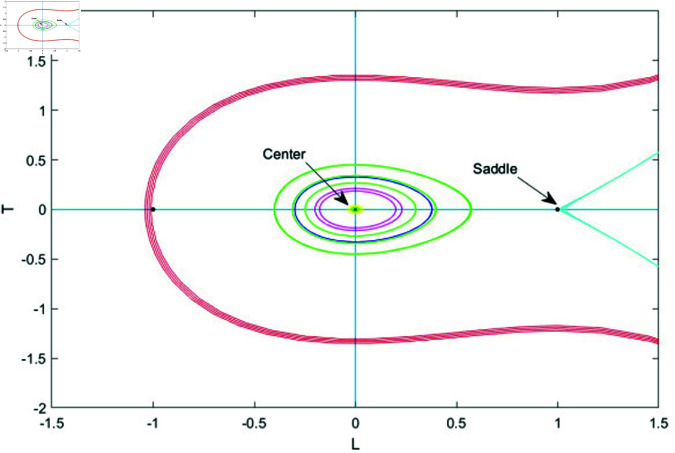
Graphical visualization of case 4 under the parametric value k = 1, b = 1 and ω=2, *s*_1_ = 3.

In case 4, system (70) has two equilibrium points: *G*_1_ = (0,0) and *G*_2_ = (1,0). Here, *G*_1_ is the center point, while *G*_2_ acts as the saddle point. The phase plots for this case are shown in Fig 15.

### 7.2 chaotic behavior analysis

Chaos behavior analysis studies the unpredictable and highly sensitive behavior in nonlinear dynamical systems. This type of analysis identifies and characterizes chaotic motion, where small variations in initial conditions lead to significantly different outcomes, making long-term prediction nearly impossible. Key tools in chaos behavior analysis include strange attractors, which visualize chaotic states; bifurcation diagrams, which show how changes in parameters lead to the onset of chaos; and Lyapunov exponents, which measure sensitivity to initial conditions. Through these methods, chaos analysis provides insights into the complex and seemingly random behavior in physical, biological, and engineered systems [41].

#### 7.2.1 Lyapunov exponent.

Lyapunov exponents are essential for identifying chaos in dynamical systems, as they measure how sensitive a system is to initial conditions. When at least one Lyapunov exponent is positive, it signals that small differences in initial states will grow exponentially, leading to unpredictable behavior—a hallmark of chaos. Positive exponents thus confirm chaotic dynamics. In this research, the nonlinear ordinary differential equations (NLODEs) derived from Eq 6 are restructured into a dynamical system as represented in Eq 76. Introducing a perturbation term into Eq 76, we analyze the resulting 2D and 3D chaotic dynamics of the system.

$$\left\{ \begin{array}{c} \frac{dU}{d\xi }=L,\\ \frac{dL}{d\xi }=N_{1}u-N_{2}u^{2}+\epsilon \cos(\phi \xi )=T, \end{array}\right.$$ (77)

**Fig 16:**
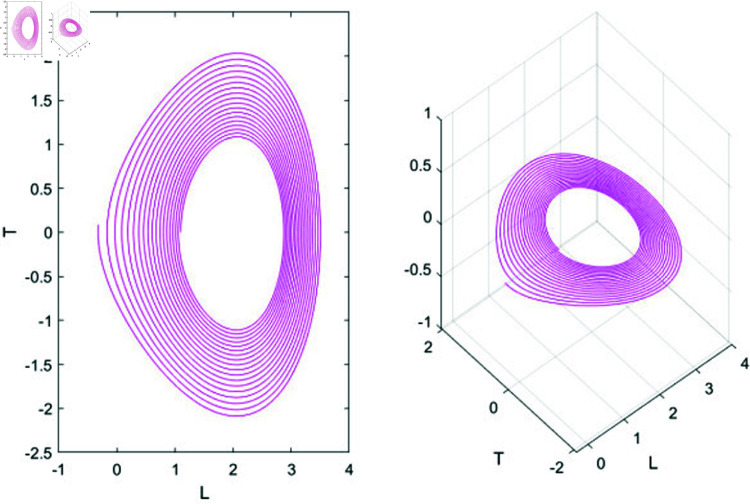
Physical illustration the 2D and 3D chaotic behavior of system (77) under specific parameter values *N*_1_ = 0.5, *N*_2_ = 0.5, ϵ=0.5, ϕ=0.1.

**Fig 17:**
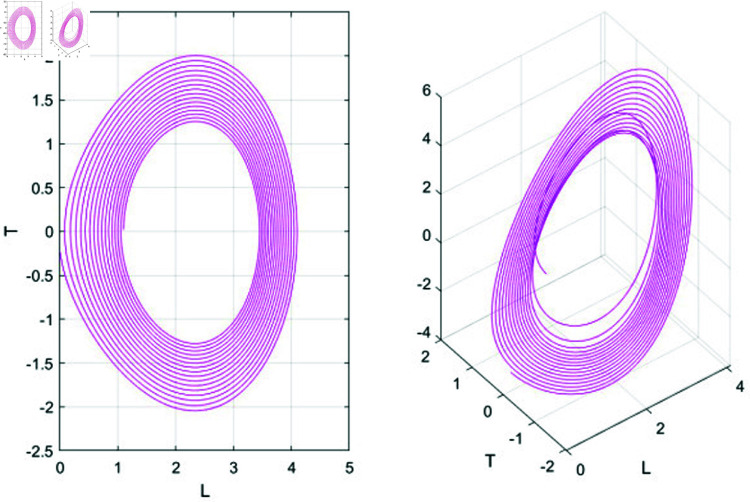
Physical illustration the 2D and 3D chaotic behavior of system (77) under specific parameter values *N*_1_ = 0.5, *N*_2_ = 0.2, ϵ=1.1, ϕ=0.7.

In this study, *ϕ* represents the frequency, while ϵ denotes the amplitude. We will investigate the impact of ϕ and ϵ on the governing model. The chaotic behavior of the system is demonstrated through both 3-D and 2-D visualizations, with various values of ϕ, ϵ, and other relevant parameters. These visualizations are depicted in Figs 16 and 17.

#### 7.2.2 Time series behavious.

**Fig 18:**
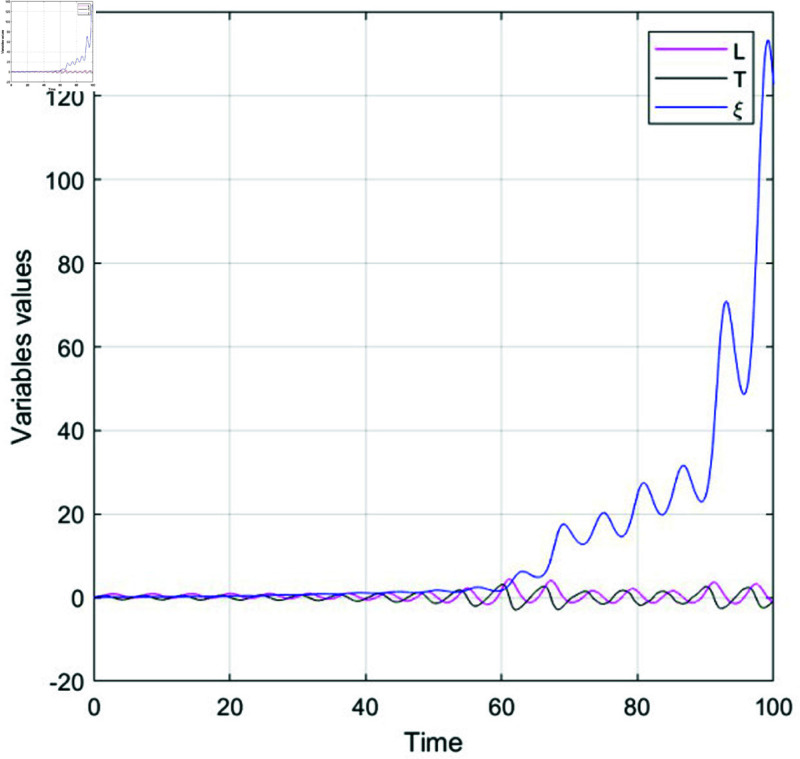
Physical visualization of time series behavior of system (77), at initial condition 0.01, 0.1, 0.1, illustrate different color curves respectively with *N*_1_ = 0.3, *N*_2_ = 0.3, ϵ = 0.5, $\phi =-\frac{\pi }{2}$.

**Fig 19:**
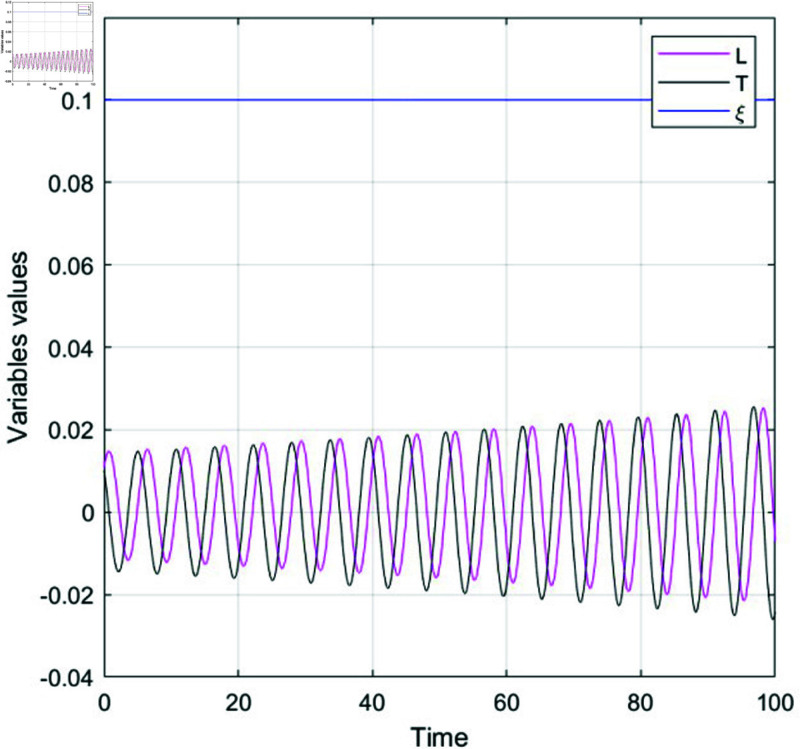
Physical visualization of time series behavior of system (77), at initial condition 0.1, 0.01, 0.1, illustrate different color curves respectively and the parametric values are *N*_1_ = 0.03, *N*_2_ = 0.07, ϵ = 0.001, $\psi =\frac{\pi }{2}$.

**Fig 20:**
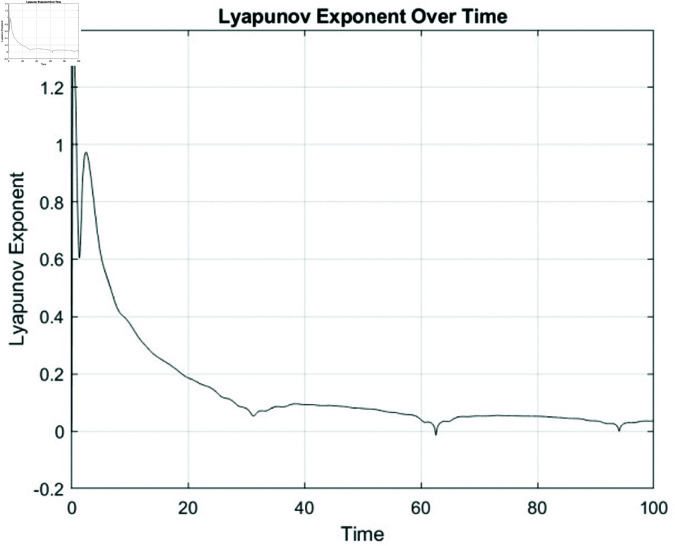
A physical interpretation of the Lyapunov exponent highlights the presence of chaos when certain parameter values are applied *N*_1_ = 0.9, *N*_2_ = 0.8, ϵ = 0.01, ϕ=π.

Time series analysis involves studying data collected over time to uncover trends, patterns, and relationships. It is frequently used to understand the behavior of state variables in dynamical systems as they change over time. This analysis helps identify recurring trends and cyclic variations. A crucial aspect is ensuring stationarity, meaning the statistical properties of the data, like mean and variance, stay consistent. Key methods in time series analysis include autocorrelation, which assesses the similarity of data points over time, and spectral analysis, which decomposes the time series into frequency components to describe system behavior through mathematical equations. The time series behavior of the proposed model is effectively illustrated through Figs 18, 19, and 20, showcasing its dynamic evolution under varying initial conditions and parameter settings.

## 8 Comparative examination

A comprehensive comparative analysis has been conducted to evaluate the solutions obtained in this study against those previously reported in the literature. In 2024, Md. Ashik Iqbal and his team utilized the $\left(G^{\prime }/(G^{\prime }+G+A)\right)$-expansion method incorporating the beta derivative, successfully identifying kink and bell-shaped solitons [16]. Similarly, Marwa M. Alzubaidi (2024) employed the Improved Modified Extended Tanh-Function Technique with Jumarie’s Modified Riemann–Liouville derivative to uncover various periodic solitons within a comparable model [18]. In contrast, our study introduces a more extensive range of soliton structures, encompassing bright, dark, singular, periodic, kink, and anti-kink waves, significantly expanding the solution space. Through our approach, we have derived a total of 59 distinct solutions and thoroughly investigated soliton behavior for varying values of α. Additionally, our study delves into the bifurcation and chaotic dynamics of the model, providing a critical stability assessment that enhances the understanding of its dynamical nature. These novel findings contribute meaningfully to the field of mathematical physics, offering deeper insights into complex wave behaviors and enriching the theoretical foundation for future studies.

## 9 Conclusion

In this article, we present a dynamical analysis of the space-time fractional Boussinesq equation, demonstrating the effectiveness of the Modified Sardar sub-equation method and the new extended direct algebraic technique. These methods, along with Atangana’s beta derivative, lead to a wide array of wave solutions. Our comprehensive analysis investigates various soliton types, including kink, anti-kink, dark, bright, periodic, and singular solitary waves, and delves deeper into the model’s dynamics by exploring bifurcation and chaos phenomena. A variety of solution forms rational,trigonometric, exponential and hyperbolic were successfully derived, highlighting the flexibility of the methods employed. A comparative analysis showed that the Modified Sardar sub-equation method yields more versatile solutions than the new extended direct algebraic method, although the latter generates a larger number of solutions. The study also explored the impact of the fractional derivative parameter on the system’s behavior, revealing significant variations based on different values of α. 2D and 3D visualizations further confirm the accuracy and relevance of the derived results. Overall, the effectiveness of the applied methods demonstrates their potential to tackle a wide range of nonlinear fractional partial differential equations in mathematical physics and other scientific fields. All computational tasks were efficiently carried out using MATHEMATICA, while phase plane analysis was conducted with MATLAB. Future research could focus on applying additional techniques, such as sensitivity analysis, to gain a deeper understanding of the model’s behavior. Additionally, exploring alternative analytical and numerical methods could further validate the results and extend the applicability of the study to more complex physical systems. We can further improve this study by using different fractional derivatives on the same model. This would help us understand its behavior better, discover new properties, and make the results more accurate.

## References

[1] Alqahtani AM, Akram S, Ahmad J, Aldwoah KA, ur Rahman M. Stochastic wave solutions of fractional Radhakrishnan–Kundu–Lakshmanan equation arising in optical fibers with their sensitivity analysis. J Opt. 2024:1–23. doi: 10.1007/s12596-024-01850-w

[2] Bates DM, Chambers JM. Nonlinear models. Statistical models in S. Routledge. 2017. p. 421–54. doi: 10.1201/9780203738535-10

[3] Ahmad J, Younas T. Wave structures of the (3+1)-dimensional nonlinear extended quantum Zakharov–Kuznetsov equation: analytical insights utilizing two high impact methods. Opt Quant Electron. 2024;56(5):882. doi: 10.1007/s11082-024-06691-2

[4] Ahmad J, Younas T. Dynamical behavior of soliton solutions to the fractional phi-four model via two analytical techniques. Mod Phys Lett B. 2024;38(32):2450310. doi: 10.1142/s021798492450310x

[5] Ahmad J, Younas T. A comprehensive study of the conformable time-fractional coupled Konno–Oono equation: new methodologies and stability analysis in magnetic field. Opt Quant Electron. 2024;56(5):1–32. doi: 10.1007/s11082-024-06752-6

[6] Younas T, Ahmad J. Dynamical behavior of the higher-order cubic-quintic nonlinear Schrödinger equation with stability analysis. J Opt. 2024:1–23. doi: 10.1007/s12596-024-01864-4

[7] Ahmad J, Younas T. Diverse optical wave structures to the time-fractional phi-four equation in nuclear physics through two powerful methods. Opt Quant Electron. 2024;56(4):606. doi: 10.1007/s11082-023-06190-w

[8] Younas T, Ahmad J. Novel soliton insights into generalized fractional Tzitz´eica-type evolution equations using the modified Khater method. Mod Phys Lett B. 2024:2450441. doi: 10.1142/S021798492450441X

[9] Labade MB. An overview of definitions of Riemann-Liouvilles fractional derivative and caputos fractional derivative. IJSR. 2021;10(4):1210–2. doi: 10.21275/sr21419125726

[10] Sikora B. Remarks on the Caputo fractional derivative. Minut. 2023;5:76–84. https://minut.polsl.pl/articles/B-23-001.pdf

[11] Kareem AM. Conformable Fractional Derivatives and It Is Applications for Solving Fractional Differential Equations. IOSR JM. 2017;13(02):81–7. doi: 10.9790/5728-1302028187

[12] Akrami MH, Owolabi KM. On the solution of fractional differential equations using Atangana’s beta derivative and its applications in chaotic systems. Sci Afr. 2023;21:e01879. doi: 10.1016/j.sciaf.2023.e01879

[13] Bona T, Chen M, Saut JC. Boussinesq equations and other systems for small-amplitude long waves in nonlinear dispersive media. I: derivation and linear theory. J Nonl Sci. 2002;12:283–318. doi: 10.1007/s00332-002-0466-4

[14] Bona JL, Chen M, Saut J-C. Boussinesq equations and other systems for small-amplitude long waves in nonlinear dispersive media: II. The nonlinear theory. Nonlinearity. 2004;17(3):925–52. doi: 10.1088/0951-7715/17/3/010

[15] El-Wakil SA, Abulwafa EM. Formulation and solution of space–time fractional Boussinesq equation. Nonl Dyn. 2014;80(1–2):167–75. doi: 10.1007/s11071-014-1858-3

[16] Iqbal MdA, Akbar MA, Islam MdA. The nonlinear wave dynamics of fractional foam drainage and Boussinesq equations with Atangana’s beta derivative through analytical solutions. Results Phys. 2024;56:107251. doi: 10.1016/j.rinp.2023.107251

[17] Razzaq W, Zafar A, Nazir A, Junjua M-D, Awwad FA, Ismail EAA. Optical soliton solutions of nonlinear differential Boussinesq water wave equation via two analytical techniques. Results Phys. 2024;64:107898. doi: 10.1016/j.rinp.2024.107898

[18] Alzubaidi MM, Almatrafi MB. New soliton solutions to the space-time fractional boussinesq equation using a reliable method. Int J Anal Appl. 2024;22:135. doi: 10.28924/2291-8639-22-2024-135

[19] Vivas-Cortez M, Arshed S, Perveen Z, Sadaf M, Akram G, Rehan K, et al. Analysis of perturbed Boussinesq equation via novel integrating schemes. PLoS One. 2024;19(5):e0302784. doi: 10.1371/journal.pone.0302784 38758758 PMC11101042

[20] Chen H, Zhu Q, Qi J. Further results about the exact solutions of conformable space–time fractional Boussinesq equation (FBE) and breaking soliton (Calogero) equation. Results Phys. 2022;37:105428. doi: 10.1016/j.rinp.2022.105428

[21] Liao S. A buoyancy vertical transport system of deep-sea mining. J Ocean Eng Sci. 2020;5(3):294–5. doi: 10.1016/j.joes.2020.05.003

[22] Hosseini K, Ansari R. New exact solutions of nonlinear conformable time-fractional Boussinesq equations using the modified Kudryashov method. Waves Random Complex Media. 2017;27(4):628–36. doi: 10.1080/17455030.2017.1296983

[23] Ali Akbar M, Akinyemi L, Yao S-W, Jhangeer A, Rezazadeh H, Khater MMA, et al. Soliton solutions to the Boussinesq equation through sine-Gordon method and Kudryashov method. Results Phys. 2021;25:104228. doi: 10.1016/j.rinp.2021.104228

[24] Ibrahim S, Ashir AM, Sabawi YA, Baleanu D. Realization of optical solitons from nonlinear Schrödinger equation using modified Sardar sub-equation technique. Opt Quant Electron. 2023;55(7):617. doi: 10.1007/s11082-023-04776-y

[25] Murad MAS, Faridi WA, Iqbal M, Arnous AH, Shah NA, Chung JD. Analysis of Kudryashov’s equation with conformable derivative via the modified Sardar sub-equation algorithm. Results Phys. 2024;60:107678. doi: 10.1016/j.rinp.2024.107678

[26] Kamel NM, Ahmed HM, Rabie WB. Retrieval of soliton solutions for 4th-order (2+1)-dimensional Schrödinger equation with higher-order odd and even terms by modified Sardar sub-equation method. Ain Shams Eng J. 2024;15(7):102808. doi: 10.1016/j.asej.2024.102808

[27] Murad MAS, Ismael HF, Sulaiman TA. A class of optical solutions for time-fractional perturbed Fokas–Lenells equation via a modified Sardar sub-equation approach. Opt Quant Electron. 2024;56(7):1–16. doi: 10.1007/s11082-024-06494-5

[28] Ali A, Ahmad J, Javed S, Rehman S-U-. Analysis of chaotic structures, bifurcation and soliton solutions to fractional Boussinesq model. Phys Scr. 2023;98(7):075217. doi: 10.1088/1402-4896/acdcee

[29] Vahidi J, Zabihi A, Rezazadeh H, Ansari R. New extended direct algebraic method for the resonant nonlinear Schrödinger equation with Kerr law nonlinearity. Optik. 2021;227:165936. doi: 10.1016/j.ijleo.2020.165936

[30] Gao W, Rezazadeh H, Pinar Z, Baskonus HM, Sarwar S, Yel G. Novel explicit solutions for the nonlinear Zoomeron equation by using newly extended direct algebraic technique. Opt Quant Electron. 2020;52(1):1–13. doi: 10.1007/s11082-019-2162-8

[31] Rehman HU, Hassan MU, Saleem MS, Nasri R, Santina D, Mlaiki N. Soliton solutions of Zakhrov equation in ionized plasma using new extended direct algebraic method. Results Phys. 2023;46:106325. doi: 10.1016/j.rinp.2023.106325

[32] Faridi WA, Myrzakulova Z, Myrzakulov R, Akgül A, Osman MS. The construction of exact solution and explicit propagating optical soliton waves of Kuralay equation by the new extended direct algebraic and Nucci’s reduction techniques. Int J Model Simul. 2024:1–20. doi: 10.1080/02286203.2024.1234567

[33] Tipu GH, Faridi WA, Myrzakulova Z, Myrzakulov R, AlQahtani SA, AlQahtani NF, et al. On optical soliton wave solutions of non-linear Kairat-X equation via new extended direct algebraic method. Opt Quant Electron. 2024;56(4):655. doi: 10.1007/s11082-024-06369-9

[34] Khalil R, Al Horani M, Yousef A, Sababheh M. A new definition of fractional derivative. J Comput Appl Math. 2014;264:65–70. doi: 10.1016/j.cam.2014.01.002

[35] Jari R, Mu L. Superconvergence of H(div) finite element approximations for the Stokes problem by local L2-projection methods. J Comput Appl Math. 2015;278:278–92. doi: 10.1016/j.cam.2014.10.008

[36] Atangana A, Baleanu D, Alsaedi A. New properties of conformable derivative. Open Math. 2015;13(1). doi: 10.1515/math-2015-0081

[37] Lavrova S, Kudryashov N. Suppression of chaos in the periodically perturbed generalized complex Ginzburg–Landau equation by means of parametric excitation. Opt Quant Electron. 2023;55(12):1114. doi: 10.1007/s11082-023-05194-w

[38] Riaz MB, Kazmi SS, Jhangeer A, Martinovic J. Unveiling solitons and dynamic patterns for a (3+1)-dimensional model describing nonlinear wave motion. MATH. 2024;9(8):20390–412. doi: 10.3934/math.2024992

[39] Qi J, Cui Q, Zhang L, Sun Y. Solution structures of an electrical transmission line model with bifurcation and chaos in hamiltonian dynamics. Int J Bifurcation Chaos. 2023;33(09):2350108. doi: 10.1142/s0218127423501080

[40] Sun Y, Qi J, Cui Q. Analyzing the occurrence of bifurcation and chaotic behaviors in multi-fractional-order stochastic ginzburg–landau equations. Fractals. 2024;32(06):1–24. doi: 10.1142/s0218348x24501056

[41] Faridi WA, Iqbal M, Ramzan B, AlQahtani SA, Osman MS, Akinyemi L, et al. The formation of invariant optical soliton structures to electric-signal in the telegraph lines on basis of the tunnel diode and chaos visualization, conserved quantities: lie point symmetry approach. Optik. 2024;305:171785. doi: 10.1016/j.ijleo.2024.171785

[42] Mirhosseini-Alizamini SM, Rezazadeh H, Eslami M, Mirzazadeh M, Korkmaz A. New extended direct algebraic method for the Tzitzica type evolution equations arising in nonlinear optics. Comput Methods Differ Equ. 2020;8(1):28–53. doi: 10.22034/cmde.2019.9472

